# Modeling HLA associations with EBV‐positive and ‐negative Hodgkin lymphoma suggests distinct mechanisms in disease pathogenesis

**DOI:** 10.1002/ijc.29467

**Published:** 2015-02-20

**Authors:** Paul C.D. Johnson, Karen A. McAulay, Dorothy Montgomery, Annette Lake, Lesley Shield, Alice Gallagher, Ann‐Margaret Little, Anila Shah, Steven G.E. Marsh, G. Malcolm Taylor, Ruth F. Jarrett

**Affiliations:** ^1^ Boyd Orr Centre for Population and Ecosystem Health, Institute of Biodiversity, Animal Health and Comparative Medicine, University of GlasgowGlasgowUnited Kingdom; ^2^ MRC ‐ University of Glasgow Centre for Virus Research, Institute of Infection, Immunity and Inflammation, College of Medical, Veterinary and Life Sciences, University of GlasgowGlasgowUnited Kingdom; ^3^ Histocompatibility and Immunogenetics Laboratory, Gartnavel General HospitalGlasgowUnited Kingdom; ^4^ Anthony Nolan, Royal Free HospitalHampsteadLondonUnited Kingdom; ^5^ Cancer Institute, University College London, Royal Free CampusLondonUnited Kingdom; ^6^ Immunogenetics GroupUniversity of Manchester, St Mary's HospitalManchesterUnited Kingdom

**Keywords:** Hodgkin lymphoma, EBV, HLA

## Abstract

HLA genotyping and genome wide association studies provide strong evidence for associations between Human Leukocyte Antigen (HLA) alleles and classical Hodgkin lymphoma (cHL). Analysis of these associations is complicated by the extensive linkage disequilibrium within the major histocompatibility region and recent data suggesting that associations with EBV‐positive and EBV‐negative cHL are largely distinct. To distinguish independent and therefore potentially causal associations from associations confounded by linkage disequilibrium, we applied a variable selection regression modeling procedure to directly typed HLA class I and II genes and selected SNPs from EBV‐stratified patient subgroups. In final models, HLA‐A*01:01 and B*37:01 were associated with an increased risk of EBV‐positive cHL whereas DRB1*15:01 and DPB1*01:01 were associated with decreased risk. Effects were independent of a prior history of infectious mononucleosis. For EBV‐negative cHL the class II SNP rs6903608 remained the strongest predictor of disease risk after adjusting for the effects of common HLA alleles. Associations with “all cHL” and differences by case EBV status reflected the subgroup analysis. In conclusion, this study extends previous findings by identifying novel HLA associations with EBV‐stratified subgroups of cHL, highlighting those alleles likely to be biologically relevant and strengthening evidence implicating genetic variation associated with the SNP rs6903608.

## Abstract 2

**What's new?**

Strong evidence exists for associations between HLA alleles and classical Hodgkin lymphoma (cHL). Analysis is however complicated by the linkage disequilibrium within the MHC region and data suggesting that associations with Epstein‐Barr virus (EBV)‐positive and negative cHL are distinct. In the largest study to date to investigate associations between EBV‐stratified cHL subgroups and directly typed HLA alleles, the authors extend associations with EBV‐positive cHL to novel HLA class II alleles, which are associated with decreased disease risk. For EBV‐negative disease, the class II SNP rs6903608 remains the strongest predictor of risk after adjusting for the effects of common HLA alleles.

**Abbreviations**
cHL: classical Hodgkin lymphoma
CI: confidence interval
CTL: cytotoxic T‐lymphocyte
EBNA: EBV nuclear antigen
EBV: Epstein‐Barr virus
EBER: Epstein‐Barr virus‐encoded small RNA
GWAS: genome wide association study or studies
HLA: human leukocyte antigen
HRS: Hodgkin and Reed‐Sternberg
IM: infectious mononucleosis
LD: linkage disequilibrium
LMP: latent membrane protein
MHC: major histocompatibility complex
OR: odds ratio
PPA: posterior probability of association
SNEHD: Scotland and Newcastle Epidemiological study of Hodgkin lymphoma
SNP: single nucleotide polymorphism

Classical Hodgkin lymphoma (cHL) is a B‐cell derived malignancy, which is one of the most common cancers of young adults.^1^, ^2^ It is unusual in that the tumor cells, the Hodgkin and Reed‐Sternberg (HRS) cells, constitute only a small fraction of the tumor mass, which is dominated by a mixed cellular infiltrate containing a high proportion of T‐cells.^3^ Interactions between HRS cells and the microenvironment are thought to play a major role in disease pathogenesis.^3^ Epstein‐Barr virus (EBV) is causally associated with approximately one third of cases in socioeconomically developed countries.^4^, ^5^ In EBV‐positive cases, HRS cells express a group of EBV latent antigens comprising EBV nuclear antigen (EBNA1) and latent membrane protein (LMP) 1, 2A and 2B.^4^ Whilst these proteins have a plausible role in disease pathogenesis,^4^ they elicit only weak cytotoxic T‐cell (CTL) responses.^6^ In a small proportion of patients, EBV‐positive cHL occurs following infectious mononucleosis (IM), a disease associated with delayed infection by EBV.^7^^−^ 9

Susceptibility to Hodgkin lymphoma was first associated with Human Leukocyte Antigen (HLA) genes within the Major Histocompatibility Complex (MHC) in the 1960 s^10^; however, associations with HLA alleles, including HLA‐A1,^11^ proved difficult to verify due to small sample sizes and low‐resolution typing methods. Subsequently, the development of molecular typing methods revealed associations with the HLA class II alleles HLA‐DPB1*02:01 and HLA‐DPB1*03:01.^12^^−^ 16 Klitz *et al*. (1994) also described links between the nodular sclerosis subtype of cHL and multiple class II haplotypes including DRB1*15:01‐DQA1*01:02‐DQB1*06:02.^17^ Within the last decade, several studies have shown that HLA‐A*01:01 and A*02:01 are associated with an increased and decreased risk of EBV‐positive cHL, respectively.^18^^−^ 21 Recently, Huang *et al*. (2012) described associations between EBV‐positive cHL and HLA‐B37 and DR10, but suggested that these were most probably due to linkage disequilibrium (LD) with HLA‐A1 on the haplotype HLA‐A1‐B37‐DR10.^22^ They also reported that DR2 (the serotype that includes DRB1*15) and DR5 were associated with an increased risk and DR7 with a decreased risk of EBV‐negative cHL.^22^ However, because their analysis used allele frequencies rather than individual genotypes they could not separate potentially causative associations from confounding due to LD in a mutually adjusted analysis. In addition, case: control analysis of DPB1 alleles was not performed.

Genome‐wide association studies (GWAS) have revealed striking associations between cHL and single nucleotide polymorphisms (SNPs) in the MHC region, with the class II SNP rs6903608 showing the strongest association with disease.^23^^−^ 26 In the only GWAS to stratify patients by EBV status in the discovery analysis, five MHC SNPs were independently associated with cHL.^24^ These included: two class I SNPs that were associated with EBV‐positive cHL and accounted for by effects of A*01:01 and A*02:01; rs6903608, a class II SNP, which was associated with EBV‐negative cHL; and rs2248462 and rs2395185 which did not show heterogeneity by EBV status. Moutsianas *et al*. (2011) analysed HLA‐A, C, B, DRB1, DQA1 and DQB1 alleles imputed from their GWAS data, but did not stratify patients by EBV status.^27^ In unconditional analyses, an increased risk of cHL was associated with DRB1*15:01, DQB1*06:02 and DQB1*03:03 and a decreased risk with DRB1*07:01 and DQA1*02:01; however, the effect of rs6903608 could not be explained by these alleles and they concluded that rs6903608, DQA1*02:01 and the DPB1 SNP rs2281389 were the main independent contributors to disease risk.

The above studies show clear associations between MHC polymorphisms and risk of cHL but, as yet, no single study has performed a mutually adjusted analysis of both HLA alleles and SNPs with cases stratified by EBV status. The aim of this study was to identify alleles likely to have a biological role in cHL pathogenesis by applying a variable selection modeling procedure to directly typed HLA class I and II genes, including DPB1, from EBV‐stratified subgroups of patients. The three SNPs that contribute independently to disease risk, but have not been accounted for by effects of HLA alleles, were also included in these analyses.

## Material and Methods

### Participants

The cHL patients included in this analysis have been described previously^21^ and were from two epidemiological studies and a case series (Table 1 and Supporting Information Table S2).^5^, ^7^, ^9^ Controls were mainly from the SNEHD study (Table 1).^9^ All cases and controls were resident in Scotland or the north of England at the time of diagnosis or recruitment, respectively.^9^ Participants were included if sufficient germline DNA was available for HLA typing and cases were included only if EBV status of tumors, defined by EBV‐encoded small RNA (EBER) *in situ* hybridisation or LMP1 immunohistochemistry, was known. The final study included 503 patients (155 EBV‐positive, 348 EBV‐negative) and 347 controls. Self‐reported history of IM was available for 97% of controls and 60% of patients.^7^, ^9^ Ethical approval was obtained from Research Ethics Committees and all participants provided informed consent.

**Table 1:** Numbers of controls and cases by sex, age, histological subtype, history of IM and study

		Controls *N* (%)	All cases *N* (%)	EBV+ve cases *N* (%)	EBV‐ve cases *N* (%)
Total participants		347	503	155	348
Sex	Female	144 (41)	225 (45)	49 (32)	176 (51)
	Male	203 (59)	278 (55)	106 (68)	172 (49)
Age group (years)	15‐34	131 (38)	272 (54)	61 (39)	211 (61)
	35‐49	81 (23)	99 (20)	31 (20)	68 (20)
	≥50	135 (39)	132 (26)	63 (41)	69 (20)
Histological subtype	Mixed cellularity		115 (23)	63 (41)	52 (15)
	Nodular sclerosis		353 (70)	79 (51)	274 (79)
	Other		35 (7)	13 (8)	22 (6)
Self‐reported IM	Not recorded	12	201	70	131
	No	316 (94)	268 (89)	72 (85)	196 (90)
	Yes	19 (6)	34 (11)	13 (15)	21 (10)
Study	SNEHD	320 (92)	283 (56)	82 (53)	201 (58)
	YHHCCS	27 (8)	41 (8)	10 (6)	31 (9)
	ITCH	0 (0)	179 (36)	63 (41)	116 (33)

All cases, all cHL cases; EBV+ve cases, EBV‐positive cHL cases; EBV‐ve cases, EBV‐negative cHL cases; N, number; IM, infectious mononucleosis; SNEHD, Scotland and Newcastle Epidemiological study of Hodgkin's Disease; YHHCCS, Young adult Hodgkin disease and Haematological malignancy Case Control Study; ITCH, Investigation of The Cause of Hodgkin lymphoma, a cHL case series.

### HLA typing and genotyping

Intermediate‐resolution typing of HLA‐A, C, B and DRB1 genes was performed on all participants (hereafter referred to as the larger dataset) at Anthony Nolan using locus‐specific PCR followed by sequence specific oligonucleotide hybridization (One Lambda, Canoga Park, CA). This generates a list of possible alleles, including common, well‐defined and rare alleles, which differ in the second field of the allele descriptor; the most likely common allele was assigned, unless stated otherwise. HLA‐DQA1, DQB1 and DPB1 typing was performed at an earlier time‐point in GMT's laboratory on participants in the SNEHD study (smaller dataset), as previously described^15^, ^28^^−^ 30 (Table 2). Genotyping results at SNPs rs6903608, rs2248462 and rs2395185 were available for >90% of individuals from previous GWAS.^23^, ^24^

**Table 2:** Number of cases and controls typed at each HLA locus

	Controls *N* (%)	All cases *N* (%)	EBV+ve cases *N* (%)	EBV−ve cases *N* (%)
Total	347	503	155	348
HLA‐A	347 (100%)	502 (99.8%)	154 (99%)	348 (100%)
HLA‐C	347 (100%)	499 (99%)	154 (99%)	345 (99%)
HLA‐B	347 (100%)	500 (99%)	154 (99%)	346 (99%)
HLA‐DRB1	311 (90%)	469 (93%)	144 (93%)	325 (93%)
HLA‐DQA1	313 (90%)	253 (50%)	75 (48%)	178 (51%)
HLA‐DQB1	308 (89%)	246 (49%)	71 (46%)	175 (50%)
HLA‐DPB1	305 (88%)	246 (49%)	73 (47%)	173 (50%)
rs6903608	341 (98%)	475 (94%)	146 (94%)	329 (95%)
rs2248462	322 (93%)	464 (92%)	141 (91%)	323 (93%)
rs2395185	339 (98%)	473 (94%)	147 (95%)	326 (94%)

Complete typing data for HLA‐A, C, B and DRB1 were available for 311 controls and 469 cases (larger dataset) and complete data for A, C, B, DRB1, DQA1, DQB1 and DPB1 were available for 287 controls and 225 cases (smaller dataset). All cases, all cHL cases; EBV+ve cases, EBV‐positive cHL cases; EBV‐ve cases, EBV‐negative cHL cases; *N*, number.

### Statistical analysis

All alleles with frequency ≥5% in any group (controls, EBV‐positive cases, EBV‐negative cases) were selected for analysis. B*35:01, control allele frequency = 4.5%, was also included because of data related to EBV‐specific immune responses and DQB1*03:03, control allele frequency = 1.8%, was added because of previous associations with cHL risk.^6^, ^17^, ^27^ This resulted in a total of 44 alleles in analyses, unless otherwise stated (Supporting Information Tables S3 and S4). We assessed whether allele carrier frequencies, *i.e.,* the proportion of individuals who possess a particular allele, among controls were representative of the northern UK population by using Fisher exact tests to compare controls with blood donors from Newcastle, Leeds and Sheffield (http://www.allelefrequencies.net, *n* = ∼12,518 for HLA‐A, B and DRB1 and *n* = 7,568 for HLA‐C).^31^ Similar large datasets were not available for DQ and DPB1 alleles. *χ* ^2^ tests of deviation from Hardy‐Weinberg equilibrium (HWE) were performed on the seven HLA loci, pooling alleles with frequency < 15%, and the three MHC SNPs.

We tested the association between carrier status of each of the HLA alleles and each of the four cHL outcomes, comprising three case:control comparisons (with cases defined as “all cHL,” EBV‐positive cHL and EBV‐negative cHL) and one case:case comparison (EBV‐positive *vs*. EBV‐negative cHL). Adjustment for multiple testing used the Bonferroni method where the significance level was 0.05 divided by the effective number of tests.^32^ SNPs were tested for association with each outcome assuming an additive model.

We next tested whether HLA‐B*08:01 and B*35:01, two alleles known to elicit immunodominant EBV‐specific CTL responses, are associated with decreased risk of EBV‐positive cHL. Associations between DRB1*15:01, DQB1*06:02, DPB1*02:01 and DPB1*03:01 and “all cHL” and EBV‐stratified subgroups were also examined as these alleles have been previously associated with cHL.^12^, ^13^, ^15^^−^ 17, 27 This group of analyses was adjusted for effects of sex, age group (15–34, 35‐49, ≥50 years) and additive effects of HLA‐A*01:01 and A*02:01, since we previously reported that these differed in the EBV‐positive and negative patients in this dataset.^21^ A carrier effect (*i.e.,* possession of a particular allele) was tested initially and, where this proved significant (*p* < 0.05), additive (*i.e.,* per allele) and homozygote effects were examined; a two point drop in the corrected Akaike information criterion^33^ was considered evidence for a better fit. Findings reported by Huang *et al*. (2012) were tested in unadjusted analyses following grouping of alleles into broad HLA serotypes.^22^

### Allele selection regression modeling

The aim of this analysis was to select the subset of alleles that best predicts each of the four cHL outcomes described above. We used a Bayesian variable selection regression method^34^ that has two advantages over traditional frequentist model selection methods. First, it explores all possible candidate models, whereas only a fraction of models are assessed by the commonly used method of stepwise regression. Second, it directly estimates the probability that an allele is associated with the outcome (the posterior probability of association or PPA). A *p*‐value, by contrast, cannot be interpreted as a measure of support without consideration of power.^35^ The prior probability of each variable being associated with the outcome was set at 5%. This choice of prior was validated by estimating the false discovery rate using permutations (Supporting Information). The low prior probability of association acts analogously to a multiple testing penalty by allowing only alleles supported by the strongest evidence to be selected.^35^ A PPA ≥90% was considered to provide strong support and a PPA of 50 − 90% moderate support for association.

Models included 44 HLA alleles and the SNPs, rs6903608, rs2248462 and rs2395185. Independent variables fitted in regression models for variable selection were: sex; age group; and carrier status of each HLA allele/SNP. To allow deviation from a codominant model to be detected, homozygote effects were also included in models if at least five individuals in either comparison group were homozygous for the allele. Alleles with a PPA ≥50% were selected and refitted in a Firth logistic regression model,^36^ with adjustment for effects of sex and age group and, where EBV‐positive patients were included, the additive effects of A*01:01 and A*02:01. Model selection was run on both the larger and smaller datasets and results amalgamated as described in Supporting Information and Table S1. For completeness, modeling was also performed using only the 44 HLA alleles without inclusion of the SNPs.

Based on allele selection, the haplotypes A*01:01‐C*06:02‐B*37:01 and rs6903608C‐DRB1*15:01‐DQB1*06:02 were added to final models of EBV‐positive and EBV‐negative cHL, respectively. Haplotypes were inferred by the single‐imputation method,^37^ based on haplotype probabilities estimated using the expectation‐maximization algorithm implemented in the *haplo.stats* package^38^ for *R*.^39^ To determine whether effects of HLA alleles and IM were independent, self‐reported history of IM was added to the final logistic regression analysis of EBV‐positive cHL.

### Power analysis

We estimated that our study was sufficiently sensitive to detect positive HLA allele‐disease associations with odds ratios (ORs) in the range 2 − 2.5 or greater (0.3 − 0.5 or less for negative associations), assuming a codominant model and adjusting for multiple testing (Supporting Information).

## Results

Complete data for HLA‐A, C, B and DRB1 alleles were available for 311 controls and 469 patients (larger dataset) and complete data for A, C, B, DRB1, DQA1, DQB1 and DPB1 alleles were available for 287 controls and 225 patients (smaller dataset; Table 2). Forty‐four HLA alleles, comprising forty‐two that passed the allele‐frequency threshold for inclusion plus B*35:01 and DQB1*03:03, were analysed. Genotype and carrier frequencies of these HLA alleles and allele frequencies of the three SNPs are presented in Supporting Information Tables S3−S5. Carrier frequencies of HLA‐A, C, B and DRB1 alleles in controls did not differ significantly from those in geographically similar blood donor populations (data not shown). Carrier frequencies of DQB1 and DPB1 alleles were also similar to those reported in small datasets from the northwest of England, with the exception of DQB1*03:04 which was present at a higher frequency in the current study than in other datasets (http://www.allelefrequencies.net/; Supporting Information Table S4). Genotype frequencies among controls deviated from HWE at two of seven HLA loci, DQB1 (*p* = 0.014) and DPB1 (*p* = 0.0032) and none of the three SNP loci (Supporting Information Table S6). Since controls were drawn from a restricted geographical area and because only two loci were affected, population stratification is an unlikely explanation for this departure from HWE.

Associations between the carrier status of each of the 44 HLA alleles and each of the four cHL outcomes were tested in unadjusted analyses, using an adjusted significance threshold of 0.0015 to account for multiple testing (Table 3, Supporting Information Methods and Table S4). Increased risk of EBV‐positive cHL was associated with A*01:01 (*p* = 9.7 × 10^−9^), C*07:01 (*p* = 5.8 × 10^−6^), B*08:01 (*p* = 8.9 × 10^−8^) and DRB1*03:01 (*p* = 6.4 × 10^−5^; Table 3). Increased risk of EBV‐negative cHL was associated with C*07:02 (*p* = 0.00072), B*07:02 (*p* = 0.00023), DRB1*15:01 (*p* = 0.00083) and DQB1*06:02 (*p* = 1.6 × 10^−5^) whereas DRB1*07:01 (*p* = 0.00027) was associated with decreased risk (Table 3). Consistent with previous results, rs6903608C was associated with an increased risk of “all cHL” and EBV‐negative cHL.^23^, ^24^ rs2248462A and rs2395185T were associated with a decreased risk of “all cHL” with no evidence of heterogeneity by EBV status (Supporting Information Table S5).

**Table 3:** Significant associations between carrier frequencies of HLA alleles and “all cHL” and EBV‐stratified subgroups in unadjusted analyses

HLA allele	Cases *vs*. controls	EBV+ cases *vs*. controls	EBV− cases *vs*. controls	EBV+ cases *vs*. EBV‐ cases
	OR (CI), *p* value	OR (CI), *p* value	OR (CI), *p* value	OR (CI), *p* value
A*01:01	*1.4 (1.1, 1.8), 0.020*	**3.1 (2.1, 4.6), 9.7e‐9**	1.0 (0.7, 1.3), 0.85	**3.2 (2.2, 4.7), 4e‐9**
C*07:01	*1.5 (1.1, 2.0), 0.0045*	**2.5 (1.7, 3.6), 5.8e‐6**	1.2 (0.9, 1.7), 0.24	**2.0 (1.4, 3.0), 0.00033**
C*07:02	1.3 (1.0, 1.8), 0.055	0.7 (0.4, 1.1), 0.092	**1.7 (1.3, 2.4), 0.00072**	**0.4 (0.2, 0.6), 1.8e‐5**
C*06:02	1.0 (0.7, 1.4), 0.81	*1.6 (1.0, 2.5), 0.039*	0.7 (0.5, 1.1), 0.11	**2.2 (1.4, 3.6), 0.00081**
B*08:01	**1.6 (1.2, 2.2), 0.0014**	**3.0 (2.0, 4.4), 8.9e‐8**	1.2 (0.9, 1.7), 0.25	**2.4 (1.6, 3.6), 9e‐6**
B*07:02	*1.4 (1.0, 1.9), 0.029*	0.7 (0.4, 1.1), 0.098	**1.8 (1.3, 2.5), 0.00023**	**0.4 (0.2, 0.6), 7.3e‐6**
B*37:01	1.4 (0.7, 2.7), 0.31	*2.9 (1.4, 6.1), 0.0038*	0.8 (0.4, 1.7), 0.55	**3.7 (1.7, 8.2), 0.00073**
DRB1*03:01	*1.5 (1.1, 2.0), 0.012*	**2.3 (1.5, 3.5), 6.4e‐5**	1.2 (0.9, 1.7), 0.29	**1.9 (1.3, 2.9), 0.0014**
DRB1*15:01	1.2 (0.9, 1.7), 0.17	*0.5 (0.3, 0.8), 0.0021*	**1.7 (1.3, 2.4), 0.00083**	**0.3 (0.2, 0.4), 1.5e‐8**
DRB1*07:01	**0.5 (0.4, 0.8), 0.00075**	0.7 (0.4, 1.1), 0.15	**0.5 (0.3, 0.7), 0.00027**	1.5 (0.9, 2.5), 0.14
DQB1*06:02	*1.5 (1.1, 2.2), 0.021*	*0.3 (0.1, 0.7), 0.0020*	**2.4 (1.6, 3.5), 1.6e‐5**	**0.1 (0.1, 0.3), 1.7e‐8**

EBV+ cases: EBV‐positive cHL cases; EBV− cases: EBV‐negative cHL cases; *vs*: *versus*; OR: odds ratio; CI: confidence interval. Results in bold are significant after correction for multiple testing; results in italics have *p* values < 0.05. Alleles are included in the table only if the *p* value for one of the outcomes was significant after correction for multiple testing.

Associations between B*08:01 and B*35:01 and EBV‐positive cHL were investigated after adjusting for sex, age group and the additive effects of A*01:01 and A*02:01; results are presented in Supporting Information Table S7. B*08:01 was associated with a significantly increased risk of EBV‐positive cHL and this was best modelled as an additive or homozygote, rather than carrier, effect [OR_homozygote_ = 4.7; 95% confidence interval (CI), 1.9 − 13.1]. B*08:01 was also associated with “all cHL” (OR_homozygote_ = 4.5; 95% CI, 1.9–11.9) and there was no evidence of heterogeneity by tumor EBV status (*p* = 0.97). B*35:01 was not significantly associated with EBV‐positive cHL (OR_carrier_ = 0.7; 95% CI, 0.3–1.6).

Consistent with previous data, DRB1*15:01 was associated with an increased risk of EBV‐negative cHL and this was best modeled as an additive or homozygote effect (OR_per‐allele_ = 1.7; 95% CI, 1.3–2.3; Supporting Information Table S7). The association with EBV‐positive cHL was in the opposite direction (OR_carrier_ = 0.5; 95% CI, 0.3–0.8) and differences by EBV status were significant (*p* = 1.2 × 10^−5^)_._ Similar results were obtained for DQB1*06:02, which is in LD with DRB1*15:01 (Supporting Information Table S7). There was an increased risk of EBV‐negative cHL (OR_homozygote_ = 5.0; 95% CI, 2.5–10.3) and a decreased risk of EBV‐positive cHL (OR_carrier_ = 0.4; 95% CI, 0.1–0.8). Differences by EBV status were significant (*p* = 2.5 × 10^−6^). We did not find a statistically significant association with DPB1*02:01, although there was significant heterogeneity by tumor EBV status (*p* = 0.040). Both EBV‐positive and negative patients were more likely to carry DPB1*03:01 than controls but differences were not significant (Supporting Information Table S7).

For comparison with published data, associations between cHL and allele frequencies, expressed as broad serotypes, were investigated in unadjusted analyses (Supporting Information Table S8 and Supporting Information Fig. S1).^22^ For “all cHL,” we corroborated associations with DR2 and DR7 but significant associations with DR5 and the less common alleles B5 and B37, were not detected. For EBV‐positive patients, we confirmed an increased frequency of A1 and B37 and a decreased frequency of A2. An increased frequency of the less common DR10 allele was detected but this association was not statistically significant. For EBV‐negative patients, an increased frequency of DR2 and decreased frequency of DR7 were corroborated; there was no significant association with DR5.

### Allele selection regression modeling

HLA alleles and SNPs associated with “all cHL,” EBV‐positive cHL, EBV‐negative cHL and that distinguish EBV‐positive from EBV‐negative cHL, were identified using a Bayesian variable selection method.^34^ Analyses included 44 HLA alleles, modelled as both carrier and homozygote effects, and three SNPs. The estimated false discovery rates were 24% for PPA ≥50 and 6.0% for PPA ≥90%. Thus, 76% and 94% of the allelic effects selected at the 50% and 90% thresholds, respectively, were estimated to be true discoveries. These estimates suggest that setting the prior probability of association at 5% resulted in broadly realistic PPA estimates. Alleles with PPA ≥50% were selected and refitted in a Firth logistic regression model.

### EBV‐positive cHL

**Figure 1:**
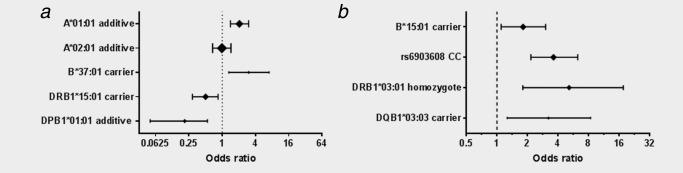
Forest plot showing final models for EBV‐positive and EBV‐negative cHL. Panel A. EBV‐positive Hodgkin lymphoma. Panel B. EBV‐negative Hodgkin lymphoma. The set of allele effects that best predicted EBV‐positive and EBV‐negative Hodgkin lymphoma cases was selected from 44 HLA alleles and three selected MHC SNPs. Effects with a posterior probability of association (PPA) ≥ 50%, estimated in a Bayesian variable selection model, were selected. The ORs and 95% CIs presented here were estimated after refitting in a Firth logistic regression model. For EBV‐positive Hodgkin lymphoma, HLA‐A*01:01 and A*02:01 were included as adjustment variables and were not subject to variable selection; analyses were adjusted for sex and age group. Symbol size reflects allele frequency.

In order of strength of association, the alleles selected by the model were DPB1*01:01 (PPA_carrier_ = 88%), A*01:01 (PPA_additive_ = 84%), B*37:01 (PPA_carrier_ = 66%) and DRB1*15:01 (PPA_carrier_ = 59%). None of the SNPs was selected, thus suggesting that their effects were accounted for by HLA alleles. Subsequent Firth logistic regression modeling indicated that A*01:01 (OR_per‐allele_ = 2.49; 95% CI, 1.75–3.59) and B*37:01 (OR = 2.58; 95% CI, 1.13–6.04) were associated with increased risk of disease whereas DRB1*15:01 (OR = 0.45; 95% CI, 0.26–0.75) and DPB1*01:01 (OR = 0.22; 95% CI, 0.06–0.65) were associated with decreased risk (Table 4, Fig. 1 *a*). Results of modeling without inclusion of the SNPs looked similar; the only qualitative difference was that DRB1*03:01 was selected in this model (PPA = 58%) but was dropped following inclusion of the SNPs (PPA = 44%) (Supporting Information Table S9).

**Table 4:** Final model for EBV‐positive Hodgkin lymphoma, adjusted for sex and age group

Variable	PPA	OR (95% CI)	*p*‐values
HLA‐A*01:01 additive#	84%	2.49 (1.75, 3.59)	2.5 × 10^−7^
HLA‐A*02:01 additive#	11%	0.97 (0.66, 1.42)	0.89
HLA‐B*37:01 carrier	66%	2.58 (1.13, 6.04)	0.024
HLA‐DRB1*15:01 carrier	59%	0.45 (0.26, 0.75)	0.0019
HLA‐DPB1*01:01 carrier◊	88%	0.22 (0.06, 0.65)	0.004

Bayesian variable selection modeling was performed on EBV‐positive Hodgkin lymphoma cases *versus* controls, with inclusion of 44 HLA alleles and three selected MHC SNPs; alleles with a posterior probability of association (PPA) ≥ 50% were selected and refitted in a Firth logistic regression model to generate ORs and CIs. #, adjustment variable, not subject to variable selection; ◊, data derived from analysis of smaller data set.

Because B*37:01 and A*01:01 are in LD, we examined whether the haplotype A*01:01‐C*06:02‐B*37:01 was associated with increased disease risk but found no evidence to support this hypothesis following adjustment for the effects of individual alleles (*p* = 0.13) (Supporting Information Table S10). Prior IM remained a significant risk factor for EBV‐positive cHL after adjusting for the effects of alleles selected in the above model (OR = 4.51; 95% CI, 1.44–14.04) (Supporting Information Table S11).

We found no evidence that A*02:01 was associated with a decreased risk of EBV‐positive cHL in allele selection modeling. There was a deficit of A*02:01‐positive carriers in EBV‐associated cases compared to controls and EBV‐negative cases (36 *vs*. 46 and 50%, respectively) but these differences were not significant after correction for multiple testing (Supporting Information Table S4). In logistic regression modeling with adjustment for the effects of sex, age group and the additive effect of A*01:01 alleles, the additive effect of A*02:01 was not significant in either the case: control or case series analyses (Supporting Information, Table S12).

### EBV‐negative cHL

There was strong support for association with the SNP rs6903608 (PPA = 95%) and moderate support for associations with DQB1*03:03 (PPA_carrier_ = 70%), DRB1*03:01 (PPA_homozygote_ = 67%) and B*15:01 (PPA_carrier_ = 60%). Associations remained significant in Firth logistic regression modeling and all were associated with increased disease risk, as presented in Table 5 and Figure 1 *b*. In modeling without inclusion of the SNPs, only DQB1*06:02 (PPA_homozygote_ = 67%) and DRB1*07:01 (PPA_homozygote_ = 64%) were selected (Supporting Information Table S13).

**Table 5:** Final model for EBV‐negative cHL, adjusted for sex and age group

Variable	PPA	OR (95% CI)	*p*‐Value
HLA‐B*15:01 carrier	60%	1.81 (1.10, 3.02)	0.020
rs6903608 C homozygote	95%	3.61 (2.16, 6.25)	4.5 × 10^−7^
HLA‐DRB1*03:01 homozygote	67%	5.14 (1.80, 17.56)	0.0018
HLA‐DQB1*03:03 carrier◊	70%	3.22 (1.26, 8.36)	0.015

Bayesian variable selection modeling was performed on EBV‐negative Hodgkin lymphoma cases *versus* controls, with inclusion of 44 HLA alleles and three selected MHC SNPs; alleles with a posterior probability of association (PPA) ≥ 50% were selected and refitted in a Firth logistic regression model to generate ORs and CIs. ◊ data derived from analysis of smaller dataset.

The C variant of rs6903608 is in LD with DRB1*15:01 (*r* ^2^ = 0.5, *D*' = 1.0) and DQB1*06:02 (*r* ^2^ = 0.4, *D*' = 0.9) and we therefore addressed whether the haplotype rs6903608C‐DRB1*15:01‐DQB1*06:02 is associated with EBV‐negative cHL. Following adjustment for variables in our model, this haplotype was not significantly associated with increased disease risk when modelled as a homozygote (*p* = 0.99) or carrier (*p* = 0.98) effect (Supporting Information Table S14).

### All cHL

The alleles and SNP selected in the modeling of all case without stratification by EBV status were DQB1*03:03 (PPA_carrier_ = 78%), rs6903608 (C_homozygote_ PPA = 74%), DRB1*03:01 (PPA_homozygote_ = 70%) and DPB1*01:01 (PPA_carrier_ = 64%), thus reflecting associations in the subgroup analyses. Further details are presented in Supporting Information Tables S15 and S16.

### EBV‐positive cHL *versus* EBV‐negative cHL

DRB1*15:01 showed the strongest evidence for heterogeneity by case group (PPA_carrier_ = 95%) followed by A*01:01 (PPA_additive_ = 77%), B*37:01 (PPA_carrier_ = 68%) and DQA1*01:02 (PPA_carrier_ = 59%; Supporting Information Table S17). DQA1*01:02 is in LD with DRB1*15:01 but associations were in opposite directions. B*27:05 also reached the threshold for selection (PPA_carrier_ = 50%) but was not significant in subsequent logistic regression modeling (OR_carrier_ = 0.47; 95% CI, 0.17–1.12). Further details are presented in Supporting Information Tables S17 and S18.

## Discussion

There is compeling evidence linking MHC polymorphisms with risk of cHL^10^, ^12^, ^13^, ^15^^−^ 21, 23 ^−^ 25, 27; however, the extensive LD within the MHC region makes it difficult to identify the causal alleles. In cHL a proportion of cases are causally associated with EBV and the available data suggest that EBV‐positive and negative cHL have distinct MHC associations, further complicating the analysis.^18^, ^20^, ^21^, ^24^ The aim of this study was to identify the HLA alleles that are most likely to independently influence cHL risk by performing allele selection regression modeling with cases stratified by EBV status. The results provide further evidence for strong HLA associations that differ by EBV status of cHL tumors.

In analyses of EBV‐positive cHL without adjustment for effects of other alleles, HLA‐A*01:01, C*07:01, B*08:01 and DRB1*03:01 were all associated with increased disease risk (Table 3). These alleles are all present on an ancestral HLA haplotype but following allele selection modeling only A*01:01 was retained in the model (Table 4 and Fig. 1 *a*). B*37:01, which has previously been associated with EBV‐positive cHL,^22^ was also selected by our model and was associated with increased disease risk. A*01:01 and B*37:01 are in LD on the haplotype A*01:01‐C*06:02‐B*37:01 but the effects of the individual alleles were independent and remained after adjusting for the effect of the haplotype (Supporting Information Table S10). Two new associations with EBV‐positive cHL were identified in this analysis. DPB1*01:01 was associated with a decreased disease risk. This allele has not been previously linked to cHL and in unadjusted analyses did not pass the significance threshold following correction for multiple testing; however, in our model DPB1*01:01 was the allele most strongly associated with EBV‐positive cHL. There was also moderately strong evidence for an association between DRB1*15:01 and decreased disease risk and this was best modelled as a carrier effect. This contrasts with the situation in EBV‐negative cHL where the additive effect of DRB1*15:01 alleles was associated with increased risk in unadjusted analyses.

Since IM is a risk factor for EBV‐positive cHL and both cHL and IM show HLA associations, prior IM was added as a variable in logistic regression analysis of alleles included in our model^7^^−^ 9, 21, 40, 41 (Supporting Information Table S11). Effects of IM remained significant indicating that HLA and IM are independent risk factors and providing no evidence for shared genetic susceptibility.

A*02:01 has previously been associated with a decreased risk of EBV‐positive cHL.^20^^−^ 22 Although A*02:01 was under‐represented in EBV‐positive cHL cases (Supporting Information Tables S3 and S4), this would be expected in an unadjusted comparison even if A*02:01 had no protective effect, because subjects carrying A*02:01 are less likely to carry A*01:01, which is a strong risk factor for EBV‐positive cHL. A*02:01 was not independently associated with EBV‐positive cHL in our adjusted analysis and gave OR estimates close to one in subsequent logistic regression analyses (Table 4 and Supporting Information Tables S9, S17 and S18). However, in a larger study that included the patients in the current study along with cases from Denmark and Sweden, A*02:01 was associated with a decreased risk of EBV‐positive cHL independently of A*01:01.^21^, ^24^ Similarly, a GWAS that included most of these cases found a significant association between EBV‐positive cHL and the A*02:01‐linked SNP rs6904029 after adjusting for the effects of the A*01:01‐linked SNP rs2734986.^24^ Neither of these studies found evidence of heterogeneity of the A*02:01 effect between the UK case series reported here and the other case series, suggesting that lack of association in our study could be due to smaller sample size. Therefore, our results do not cast doubt on the previously reported A*02:01 associations.

HLA class I and II molecules present peptides from exogenous pathogens to CD8 and CD4‐positive T‐cells, respectively; we therefore believe that HLA associations with EBV‐positive cHL are likely to reflect qualitative and/or quantitative differences in the T‐cell response to EBV. To date, no confirmed EBV epitopes or CTL responses restricted by A*01:01 have been described. Similarly, B*37:01‐restricted CTL responses to peptides from EBNA1, LMP‐1 or LMP‐2, the antigens expressed by HRS cells, have not been identified.^6^ An association between these alleles and increased disease risk is therefore plausible; however, A*01:01 is in LD with B*08:01 and there are robust EBV‐specific responses restricted through this allele.^6^ Although the immunodominant B*08:01‐restricted responses are to EBNA3A and the lytic cycle protein BZLF1,^6^ an EBNA1 epitope has also been identified.^42^, ^43^ We therefore investigated whether B*08:01 confers any protective effect after adjusting for the effects of A*01:01 (Supporting Information Table S7); consistent with Huang *et al*. (2012), we found no support for this hypothesis.^22^ Because HLA‐ B*35:01 also elicits an immunodominant EBNA1 response, we specifically included this allele in our analysis.^6^ B*35:01 carriers were less frequent among EBV‐positive patients than controls, but differences were not significant and this allele was not selected in our model. Therefore, despite consistent associations between HLA class I alleles and EBV‐positive cHL, there is currently no evidence that common alleles that elicit good CTL responses confer protection against this disease.

The identification of novel associations between EBV‐positive cHL and DPB1*01:01 and DRB1*15:01 provides evidence that HLA associations with EBV‐positive cHL extend to the class II region. Furthermore, these alleles are associated with decreased rather than increased disease risk. Class II‐restricted responses to most EBV latent proteins have been described and many are to EBNA1 epitopes.^6^ These include a DRB1*15:01‐restricted response, which could explain the association observed in this study.^44^ No DPB1*01:01‐restricted EBV responses have been identified to date, but HLA restrictions of epitopes presented by class II have been less extensively characterized than their class I counterparts. Further laboratory analyses are required to identify DRB1*15:01 and DPB1*01:01‐restricted EBV epitopes and characterise the associated effector T‐cell responses. Such studies will not only improve our understanding of the natural history of cHL but will have broader implications for T‐cell based immunotherapy.

In unadjusted analyses of EBV‐negative cHL, we confirmed previously described associations with DRB1*07:01, DRB1*15:01 and DQB1*06:02 (Table 3, Supporting Information Table S7)^17^, ^22^; however, a very different picture emerged following allele selection analysis, which adjusted for confounding among loci due to LD. In modeling without inclusion of the SNPs, DRB1*07:01 was selected along with DQB1*06:02, which was included at the expense of DRB1*15:01 (Supporting Information Table S13). After addition of the SNPs to the analysis, neither DQB1*06:02 nor DRB1*07:01 passed thresholds for selection and rs6903608 emerged as the strongest predictor of disease status (PPA = 95%; Table 5, Fig. 1 *b*). This finding is consistent with the imputation analysis described by Moutsianas *et al*. (2011).^27^ Three new alleles, B*15:01, DRB1*03:01 and DQB1*03:03, were also selected by our model and all were associated with increased risk of disease. DQB1*03:03, which is in LD with DRB1*07:01, has previously been associated with decreased rather than the increased disease risk as described here.^17^, ^27^

The rs6903608 variant is located close to HLA‐DRA and has been identified as a *cis*‐expression Quantitative Trait Locus (eQTL) for HLA‐DRA expression (http://genenetwork.nl/bloodeqtlbrowser), 45 raising the possibility that it is decreased expression of HLA‐DR, rather than any particular allele, that increases risk of EBV‐negative cHL. Down‐regulation of HLA class II on HRS cells is a characteristic feature of EBV‐negative cHL, occurring in the majority of cases.^46^ Although this is an attractive mechanism that may allow HRS cells to evade immune destruction, it remains possible that rs6903608 is in LD with another MHC gene that is the critical risk factor. Further analysis of the MHC region is required to determine the biological mechanism underlying the strong association with this SNP.

Alleles selected in modeling of “all cases” *versus* controls simply reflected those in the final models of EBV‐positive and negative disease (Supporting Information Tables S15 and S16). Similarly, alleles that best discriminated EBV‐positive from EBV‐negative disease were largely those associated with EBV‐positive cHL (Supporting Information Tables S17 and S18).

This is the largest study examining HLA associations with EBV‐stratified cHL subgroups using directly typed HLA alleles that has been performed thus far. In preliminary analyses we confirmed that our controls were representative of the northern UK population from which patients originated. In unadjusted analyses, we also validated previously reported associations with cHL including the main findings reported by Huang *et al*. (2012).^17^, ^22^ Associations with DPB1*02:01 and DPB1*03:01 were not statistically significant in this analysis although risks, at least for EBV‐negative cHL, were in the same direction as previously reported.^13^ Overall, these comparisons indicated that this was a robust dataset for further analysis, *i.e.,* allele selection modeling. However, although the study was relatively large, it included only 155 EBV‐positive cHL cases so that there was insufficient power to detect weak associations (*i.e.,* those with ORs between 0.5 and 2). Sensitivity was considerably less for DQ and DP alleles, where only half of the cases were typed (Table 2 and Supporting Information). In addition, DQB1 and DPB1 alleles in controls were not in HWE, as assessed by the genotypes of the most common alleles (Supporting Information Table S6). The cause of this is unclear but population stratification and selection appear unlikely mechanisms.^47^ These two HLA loci, along with DQBA1, were genotyped at an earlier time‐point than the other four loci and we favor the idea that some rare alleles have escaped detection, thus leading to an apparent excess of homozygotes. Carrier frequencies of DQB1 and DPB1 alleles were generally similar to those in comparable studies and identical HLA typing methods were applied to cases and controls. Deviation from HWE is therefore unlikely to have affected the overall results; nevertheless, some caution should be exercised in the interpretation of findings related to DQB1 and DPB1.

In conclusion, our data provide further evidence that the natural histories of EBV‐positive and ‐negative cHL are different, underscoring the importance of analysing EBV‐stratified subgroups in biological studies of cHL. We show that associations between EBV‐positive cHL and A*01:01 and B*37:01 are independent and identify novel associations with the class II alleles DRB1*15:01 and DPB1*01:01. For EBV‐negative cHL, we show that the class II SNP rs6903608 remains the strongest predictor of disease after adjusting for the effects of common HLA alleles.

## Supporting information

Supporting Information
