# Supplementary material for: Modeling HLA associations with EBV‐positive and ‐negative Hodgkin lymphoma suggests distinct mechanisms in disease pathogenesis
Source: Int J Cancer. 2015 Feb 20;137(5):1066–75. doi: 10.1002/ijc.29467 (PMC4737225; doi:10.1002/ijc.29467)
Supplement: Supplementary file 1 — Supporting Information [file IJC-137-1066-s001.doc]

**Supporting Information**

**Statistical analysis**

**General statistical methods**

All analysis was performed in the *R* statistical environment.^1^ All logistic regression models were fitted using Firth’s penalised-likelihood method^3^ implemented in the *logistf* package^4^ for *R* rather than maximum likelihood, because low or zero prevalence of cases or controls in any genotype class invalidates estimates of odds ratio and p-values from logistic regression models fitted using maximum likelihood.

**Hardy-Weinberg equilibrium tests**

χ^2^ tests of deviation from Hardy-Weinberg equilibrium (HWE) were performed in control subjects on the seven HLA loci and the three SNPs. HLA alleles with frequency < 15% were pooled to avoid low expected genotype counts.

**Hypothesis testing**

Hypothesis tests that compared nested models, including all tests of covariate effects, were based on profile penalized log likelihood p-values estimated from Firth logistic regression models. Comparisons of non-nested models, in particular comparison of carrier and additive inheritance models, were compared using the corrected Akaike information criterion (AICc) with the lower of the numbers of cases and controls as n^5^. A difference of at least two units indicated better fit for the model with lower AICc. Associations between carriage of individual HLA alleles or serotypes and cHL outcomes were tested in unadjusted Firth logistic regression models. All other models were adjusted for effects of sex and age group (15-34, 35-49, ≥50 years) and all models where the case definition included EBV-positive cHL patients were additionally adjusted for additive effects of HLA-A*01:01 and A*02:01.

**Allele selection regression modelling**

The aim of this analysis was to select from a large set of HLA alleles the subset that best predicts each of the four cHL outcomes, i.e. case versus control, EBV-positive case versus control, EBV-negative case versus control and EBV-positive case versus EBV-negative case. This is a variable selection problem, where the task is to select from *p* variables the subset that is independently associated with the outcome. Here, *p* is greater than the number of alleles because we tested for both carrier and homozygote effects of each allele, where numbers allowed. Forward and backward stepwise selection, although commonly used for this purpose, e.g. Morris et al (2012),^6^ has long been known to produce biased and unstable results.^7^ A particular problem when, as here, *p* is large, is that stepwise selection considers only a small number (*p* + 1) of the 2*^p^* possible subsets, leaving the great majority of the model space unexplored. Instead we used a Monte Carlo Markov Chain (MCMC) Bayesian variable selection method that simultaneously estimates all *p* regression coefficients in a single probit regression model.^8^ Variable selection is performed by specifying a prior probability, *π*, of each variable being associated with the outcome, and estimating a posterior probability of association (PPA) given the data. We set *π* = 5%, implying a high degree of scepticism, *a priori*, regarding the association of any given allelic effect with each outcome. The practical effect of imposing scepticism on non-zero regression coefficients is to force their estimates to be zero, i.e. excluded from the model, unless there is sufficiently strong evidence that they are independently associated with the outcome. Our choice of *π* was based on the reasoning that, based on known HLA-cHL associations, we would *a priori* expect only about 1 in 20 allelic effects to be associated with cHL. We classed PPA greater than 90% as strong support for association and PPA between 50% and 90% as moderate support. The MCMC sampler was run for 50,000 iterations following 5,000 burn-in iterations.

Because the choice of *π* is subjective, we objectively quantified the stringency of selection by using permutations to estimate the false discovery rate (FDR) at the 50% and 90% PPA thresholds. The FDR is the proportion of discoveries (selected variables) that are false positives (i.e. due to type I error), and was calculated as the estimated number of false positive allelic effects divided by the total number of allelic effects selected (sex and age group were excluded). The number of false positives was estimated as the mean number of allelic effects selected at each PPA threshold across 100 simulated data sets that were identical to the real data with the exception that association between the outcome and the predictors had been broken by randomly permuting the outcome vector. FDR estimates were averaged across the four cHL outcomes and the four iterations of the model selection procedure (4 HLA loci with and without SNPs and 7 HLA loci with and without SNPs). To account for sampling error, average FDR was calculated as a weighted mean of the individual FDR estimates, where the weights were the number of selected allelic effects (discoveries). For the permutation analysis the number of MCMC iterations was reduced by a factor of 10 to make FDR estimation computationally feasible.

The independent variables fitted in the regression models for variable selection were: sex, age group (15-34/35-49/50+ years) and every HLA allele that passed inclusion thresholds (Tables S3 and S4), represented as both carrier and homozygote effects. Allelic effects were included in the model if at least five subjects were represented in either of the two outcome groups being compared. This had the effect of excluding homozygote effects of rare alleles.

Because about 50% of cases were genotyped only at A, C, B and DRB1 and not at DQA1, DQB1 or DPB1 (Table 2), we ran the model selection twice, once with the larger data set and only four HLA loci, and once with all seven HLA loci but a smaller data set. Differences between these two analyses in the A, C, B and DRB1 alleles selected could be attributed to either biologically meaningful effects of adjusting for DQA1, DQB1 or DPB1 alleles or a trivial sampling artefact due to the reduced data set. To allow these two causes to be distinguished, we also ran the model selection on the smaller data set with only A, C, B and DRB1; alleles were selected for inclusion in the final model according to the rules presented in the table below.

**Table S1: Criteria used to select A, C, B and DRB1 alleles that gave conflicting results in the analysis of the larger and smaller datasets**

| Selected in 4 gene analysis using larger dataset. | Selected in 7 gene analysis using smaller dataset. | Selected in 4 gene analysis using smaller dataset. | Interpretation of  inclusion in / omission from  7 gene analysis | Include in final model |
| --- | --- | --- | --- | --- |
| Yes | No | Yes | No independent association | No |
| Yes | No | No | Sampling artefact | Yes |
| No | Yes | Yes | Sampling artefact | No |
| No | Yes | No | Independent association | Yes |

Odds ratios, 95% confidence intervals and p-values for the effects of alleles selected at the 50% threshold were estimated in Firth logistic regression models, adjusted for age group and sex, and, where EBV-positive cases were included, the additive effects of A*01:01 and A*02:01.

**Power analysis**

To gauge the sensitivity of the study, we used simulations to estimate the smallest detectable difference in carrier frequency between controls (n = 347) and the smallest case group, EBV-positive cHL (n = 155). We assumed a carrier frequency among controls of 22%, which is the average, and a higher carrier frequency among cases. The HLA-wide significance level of 0.05 was adjusted for multiple testing to 0.0015 using a method that accounts for linkage disequilibrium.^9^ We estimated that an association between carrier status and cases equivalent to an odds ratio of 2.3 (carrier frequency among cases = 39%) would give 80% power. Analyses that use higher numbers of cases will be more sensitive, with the most sensitive being between controls and all cases (n = 503), where an odds ratio of 1.9 would be detectable (carrier frequency among cases = 35%). The study is therefore sufficiently sensitive to detect minimum risk effect sizes represented by odds ratios in the range 2-2.3 (0.3-0.5 for protective alleles), approximately, which are highly plausible in the light of previous studies.^2,10^ Sensitivity to detect associations with DQ and DP alleles, where only approximately half of cases were typed, was lower, with odds ratios above 3 or below 0.13 being detectable with 80% power.

**Table S2: Numbers of controls and cases within each study by sex, age, histological subtype and history of infectious mononucleosis**

|  |  | Controls  N (%) | All cases  N (%) | EBV+ve cases  N (%) | EBV-ve cases  N (%) |
| --- | --- | --- | --- | --- | --- |
| **SNEHD** |  |  |  |  |  |
| All |  | 320 | 283 | 82 | 201 |
| Sex | Female  Male | 131 (41)  189 (59) | 128 (45)  155 (55) | 25 (30)  57 (70) | 103 (51)  98 (49) |
| Age group (years) | 15-34  35-49  ≥50 | 104 (33)  81 (25)  135 (42) | 145 (51)  57 (20)  81 (29) | 27 (33)  17 (21)  38 (46) | 118 (59)  40 (20)  43 (21) |
| Histological subtype | Mixed cellularity  Nodular sclerosis  Other |  | 64 (23)  198 (70)  21 (7.4) | 36 (44)  39 (48)  7 (8.5) | 28 (14)  159 (79)  14 (7.0) |
| Self-reported IM | Not recorded  No  Yes | 3  302 (95)  15 (5) | 20  237 (90)  26 (10) | 7  66 (88)  9 (12) | 13  171 (91)  17 (9) |
| **YHHCCS** |  |  |  |  |  |
| All |  | 27 | 41 | 10 | 31 |
| Sex | Female  Male | 13 (48)  14 (52) | 19 (46)  22 (54) | 2 (20)  8 (80) | 17 (55)  14 (45) |
| Age group (years) | 15-34 | 27 (100) | 41 (100) | 10 (100) | 31 (100) |
| Histological subtype | Mixed cellularity  Nodular sclerosis  Other |  | 9 (22)  32 (78)  0 (0) | 5 (50)  5 (50)  0 (0) | 4 (13)  27(87)  0 (0) |
| Self-reported IM | Not recorded  No  Yes | 9  14 (78)  4 (22) | 2  31 (79)  8 (21) | 0  6 (60)  4 (40) | 2  25 (86)  4 (14) |
| **ITCH** |  |  |  |  |  |
| All |  |  | 179 | 63 | 116 |
| Sex | Female  Male |  | 78 (44)  101 (56) | 22 (35)  41 (65) | 56 (48)  60 (52) |
| Age group (years) | 15-34  35-49  ≥50 |  | 86 (48)  42 (24)  51 (28) | 24 (38)  14 (22)  25 (40) | 62 (53)  28 (24)  26 (22) |
| Histological subtype | Mixed cellularity  Nodular sclerosis  Other |  | 42 (24)  123 (69)  14 (7.8) | 22 (35)  35 (56)  6 (9.5) | 20 (17)  88 (76)  8 (6.9) |
| Self-reported IM | Not reported |  | 179 | 63 | 116 |

**Case characteristics by study and case: control status, with cases further stratified by EBV status of tumours. SNEHD, Scotland and Newcastle Epidemiological study of Hodgkin’s Disease; YHHCCS, Young adult Hodgkin’s disease and Haematological malignancy Case Control Study; ITCH, Investigation of the Cause of Hodgkin lymphoma; all cases, all classical Hodgkin lymphoma cases; EBV+ve cases, EBV-positive classical Hodgkin lymphoma cases; EBV-ve cases, EBV-negative classical Hodgkin lymphoma cases; IM, infectious mononucleosis; N, number. YHHCCS was a study of young adults aged 16-24 years. ITCH is an ongoing, prospective collection of newly diagnosed Hodgkin lymphoma cases in the West of Scotland and previously also in the northern region of England. The association between prior infectious mononucleosis and risk of Hodgkin lymphoma has been reported previously for YHHCCS and SNEHD.**

| **Table S3: Genotype frequencies of common HLA alleles in cases and controls** | | | | | | | | | | | | |
| --- | --- | --- | --- | --- | --- | --- | --- | --- | --- | --- | --- | --- |
| N alleles: | Controls | | | All cases | | | EBV+ cases | | | EBV− cases | | |
|  | 0 | 1 | 2 | 0 | 1 | 2 | 0 | 1 | 2 | 0 | 1 | 2 |
| HLA-A*01:01 HLA-A*02:01 HLA-A*03:01 HLA-A*24# HLA-A*11:01 HLA-C*07:01 HLA-C*07:02 HLA-C*06:02 HLA-C*05:01 HLA-C*04:01 HLA-C*03:03 HLA-B*08:01 HLA-B*07:02 HLA-B*44:02 HLA-B*15:01 HLA-B*37:01 HLA-B*27:05 HLA-B*44:03 HLA-B*35:01 HLA-DRB1*03:01 HLA-DRB1*15:01 HLA-DRB1*04:01 HLA-DRB1*07:01 HLA-DRB1*01:01 HLA-DRB1*13:01 HLA-DQA1*05:01 HLA-DQA1*01:02 HLA-DQA1*03:01 HLA-DQA1*01:01 HLA-DQA1*02:01 HLA-DQA1*01:03 HLA-DQB1*02:01 HLA-DQB1*06:02 HLA-DQB1*05:01 HLA-DQB1*03:01 HLA-DQB1*03:02 HLA-DQB1*06:03 HLA-DQB1*03:04 HLA-DQB1*03:03 HLA-DPB1*04:01 HLA-DPB1*03:01 HLA-DPB1*02:01 HLA-DPB1*04:02 HLA-DPB1*01:01 | 222 (64%) 189 (54%) 248 (71%) 297 (86%) 312 (90%) 241 (69%) 255 (73%) 283 (82%) 258 (74%) 297 (86%) 319 (92%) 263 (76%) 258 (74%) 260 (75%) 312 (90%) 333 (96%) 309 (89%) 311 (90%) 319 (92%) 228 (73%) 215 (69%) 232 (75%) 236 (76%) 255 (82%) 279 (90%) 181 (58%) 218 (70%) 192 (61%) 221 (71%) 238 (76%) 283 (90%) 181 (59%) 227 (74%) 232 (75%) 233 (76%) 235 (76%) 278 (90%) 280 (91%) 297 (96%) 113 (37%) 250 (82%) 260 (85%) 249 (82%) 267 (88%) | 112 (32%) 121 (35%) 92 (27%) 47 (14%) 35 (10%) 96 (28%) 82 (24%) 60 (17%) 84 (24%) 47 (14%) 27 (8%) 78 (22%) 79 (23%) 81 (23%) 35 (10%) 14 (4%) 36 (10%) 34 (10%) 25 (7%) 79 (25%) 89 (29%) 73 (23%) 69 (22%) 53 (17%) 31 (10%) 112 (36%) 82 (26%) 107 (34%) 80 (26%) 68 (22%) 29 (9%) 101 (33%) 68 (22%) 65 (21%) 63 (20%) 68 (22%) 26 (8%) 25 (8%) 11 (4%) 124 (41%) 47 (15%) 41 (13%) 47 (15%) 25 (8%) | 13 (4%) 37 (11%) 7 (2%) 3 (1%) 0 (0%) 10 (3%) 10 (3%) 4 (1%) 5 (1%) 3 (1%) 1 (0%) 6 (2%) 10 (3%) 6 (2%) 0 (0%) 0 (0%) 2 (1%) 2 (1%) 3 (1%) 4 (1%) 7 (2%) 6 (2%) 6 (2%) 3 (1%) 1 (0%) 20 (6%) 13 (4%) 14 (4%) 12 (4%) 7 (2%) 1 (0%) 26 (8%) 13 (4%) 11 (4%) 12 (4%) 5 (2%) 4 (1%) 3 (1%) 0 (0%) 68 (22%) 8 (3%) 4 (1%) 9 (3%) 13 (4%) | 281 (56%) 274 (55%) 381 (76%) 424 (84%) 454 (90%) 299 (60%) 336 (67%) 410 (82%) 401 (80%) 440 (88%) 451 (90%) 328 (66%) 337 (67%) 402 (80%) 440 (88%) 472 (94%) 459 (92%) 470 (94%) 464 (93%) 304 (65%) 302 (64%) 392 (84%) 401 (86%) 403 (86%) 400 (85%) 134 (53%) 165 (65%) 176 (70%) 190 (75%) 211 (83%) 217 (86%) 142 (58%) 159 (65%) 202 (82%) 197 (80%) 209 (85%) 210 (85%) 224 (91%) 230 (93%) 87 (35%) 186 (76%) 212 (86%) 207 (84%) 229 (93%) | 177 (35%) 195 (39%) 110 (22%) 73 (15%) 48 (10%) 157 (31%) 138 (28%) 87 (17%) 94 (19%) 53 (11%) 43 (9%) 133 (27%) 136 (27%) 90 (18%) 58 (12%) 27 (5%) 41 (8%) 30 (6%) 34 (7%) 133 (28%) 136 (29%) 72 (15%) 65 (14%) 63 (13%) 67 (14%) 92 (36%) 63 (25%) 69 (27%) 58 (23%) 39 (15%) 35 (14%) 79 (32%) 56 (23%) 38 (15%) 46 (19%) 35 (14%) 33 (13%) 19 (8%) 16 (7%) 104 (42%) 48 (20%) 30 (12%) 37 (15%) 9 (4%) | 44 (9%) 33 (7%) 11 (2%) 5 (1%) 0 (0%) 43 (9%) 25 (5%) 2 (0%) 4 (1%) 6 (1%) 5 (1%) 39 (8%) 27 (5%) 8 (2%) 2 (0%) 1 (0%) 0 (0%) 0 (0%) 2 (0%) 32 (7%) 31 (7%) 5 (1%) 3 (1%) 3 (1%) 2 (0%) 27 (11%) 25 (10%) 8 (3%) 5 (2%) 3 (1%) 1 (0%) 25 (10%) 31 (13%) 6 (2%) 3 (1%) 2 (1%) 3 (1%) 3 (1%) 0 (0%) 55 (22%) 12 (5%) 4 (2%) 2 (1%) 8 (3%) | 56 (36%) 99 (64%) 120 (78%) 131 (85%) 143 (93%) 74 (48%) 124 (81%) 113 (73%) 127 (82%) 137 (89%) 144 (94%) 79 (51%) 125 (81%) 129 (84%) 144 (94%) 137 (89%) 148 (96%) 147 (95%) 146 (95%) 78 (54%) 119 (83%) 124 (86%) 118 (82%) 123 (85%) 123 (85%) 37 (49%) 59 (79%) 51 (68%) 55 (73%) 57 (76%) 67 (89%) 31 (44%) 64 (90%) 53 (75%) 56 (79%) 59 (83%) 62 (87%) 65 (92%) 66 (93%) 26 (36%) 55 (75%) 58 (79%) 64 (88%) 70 (96%) | 71 (46%) 52 (34%) 32 (21%) 23 (15%) 11 (7%) 55 (36%) 25 (16%) 40 (26%) 25 (16%) 15 (10%) 8 (5%) 51 (33%) 23 (15%) 23 (15%) 10 (6%) 16 (10%) 6 (4%) 7 (5%) 7 (5%) 47 (33%) 22 (15%) 20 (14%) 23 (16%) 21 (15%) 20 (14%) 28 (37%) 11 (15%) 23 (31%) 18 (24%) 16 (21%) 8 (11%) 28 (39%) 7 (10%) 16 (23%) 14 (20%) 12 (17%) 9 (13%) 6 (8%) 5 (7%) 32 (44%) 15 (21%) 11 (15%) 9 (12%) 3 (4%) | 27 (18%) 3 (2%) 2 (1%) 0 (0%) 0 (0%) 25 (16%) 5 (3%) 1 (1%) 2 (1%) 2 (1%) 2 (1%) 24 (16%) 6 (4%) 2 (1%) 0 (0%) 1 (1%) 0 (0%) 0 (0%) 1 (1%) 19 (13%) 3 (2%) 0 (0%) 3 (2%) 0 (0%) 1 (1%) 10 (13%) 5 (7%) 1 (1%) 2 (3%) 2 (3%) 0 (0%) 12 (17%) 0 (0%) 2 (3%) 1 (1%) 0 (0%) 0 (0%) 0 (0%) 0 (0%) 15 (21%) 3 (4%) 4 (5%) 0 (0%) 0 (0%) | 225 (65%) 175 (50%) 261 (75%) 293 (84%) 311 (89%) 225 (65%) 212 (61%) 297 (86%) 274 (79%) 303 (88%) 307 (89%) 249 (72%) 212 (61%) 273 (79%) 296 (86%) 335 (97%) 311 (90%) 323 (93%) 318 (92%) 226 (70%) 183 (56%) 268 (82%) 283 (87%) 280 (86%) 277 (85%) 97 (54%) 106 (60%) 125 (70%) 135 (76%) 154 (87%) 150 (84%) 111 (63%) 95 (54%) 149 (85%) 141 (81%) 150 (86%) 148 (85%) 159 (91%) 164 (94%) 61 (35%) 131 (76%) 154 (89%) 143 (83%) 159 (92%) | 106 (30%) 143 (41%) 78 (22%) 50 (14%) 37 (11%) 102 (30%) 113 (33%) 47 (14%) 69 (20%) 38 (11%) 35 (10%) 82 (24%) 113 (33%) 67 (19%) 48 (14%) 11 (3%) 35 (10%) 23 (7%) 27 (8%) 86 (26%) 114 (35%) 52 (16%) 42 (13%) 42 (13%) 47 (14%) 64 (36%) 52 (29%) 46 (26%) 40 (22%) 23 (13%) 27 (15%) 51 (29%) 49 (28%) 22 (13%) 32 (18%) 23 (13%) 24 (14%) 13 (7%) 11 (6%) 72 (42%) 33 (19%) 19 (11%) 28 (16%) 6 (3%) | 17 (5%) 30 (9%) 9 (3%) 5 (1%) 0 (0%) 18 (5%) 20 (6%) 1 (0%) 2 (1%) 4 (1%) 3 (1%) 15 (4%) 21 (6%) 6 (2%) 2 (1%) 0 (0%) 0 (0%) 0 (0%) 1 (0%) 13 (4%) 28 (9%) 5 (2%) 0 (0%) 3 (1%) 1 (0%) 17 (10%) 20 (11%) 7 (4%) 3 (2%) 1 (1%) 1 (1%) 13 (7%) 31 (18%) 4 (2%) 2 (1%) 2 (1%) 3 (2%) 3 (2%) 0 (0%) 40 (23%) 9 (5%) 0 (0%) 2 (1%) 8 (5%) |

N, number of; all cases, all classical Hodgkin lymphoma cases; EBV+ cases, EBV-positive classical Hodgkin lymphoma cases; EBV- cases, EBV-negative classical Hodgkin lymphoma cases. #A*24 includes A*24:02 and A*24:03.

| | **Table S4: Carrier frequencies of common HLA alleles in cases and controls** | | | | | | | | | | --- | --- | --- | --- | --- | --- | --- | --- | --- | |  | Controls | All cases | EBV+ cases | EBV− cases | Cases vs controls | EBV+ vs controls | EBV− vs controls | EBV+ vs EBV− | | HLA-A*01:01 HLA-A*02:01 HLA-A*03:01 HLA-A*24# HLA-A*11:01 HLA-C*07:01 HLA-C*07:02 HLA-C*06:02 HLA-C*05:01 HLA-C*04:01 HLA-C*03:03 HLA-B*08:01 HLA-B*07:02 HLA-B*44:02 HLA-B*15:01 HLA-B*37:01 HLA-B*27:05 HLA-B*44:03 HLA-B*35:01 HLA-DRB1*03:01 HLA-DRB1*15:01 HLA-DRB1*04:01 HLA-DRB1*07:01 HLA-DRB1*01:01 HLA-DRB1*13:01 HLA-DQA1*05:01 HLA-DQA1*01:02 HLA-DQA1*03:01 HLA-DQA1*01:01 HLA-DQA1*02:01 HLA-DQA1*01:03 HLA-DQB1*02:01 HLA-DQB1*06:02 HLA-DQB1*05:01 HLA-DQB1*03:01 HLA-DQB1*03:02 HLA-DQB1*06:03 HLA-DQB1*03:04 HLA-DQB1*03:03 HLA-DPB1*04:01 HLA-DPB1*03:01 HLA-DPB1*02:01 HLA-DPB1*04:02 HLA-DPB1*01:01 | 125/347 (36%) 158/347 (46%) 99/347 (29%) 50/347 (14%) 35/347 (10%) 106/347 (31%) 92/347 (27%) 64/347 (18%) 89/347 (26%) 50/347 (14%) 28/347 (8%) 84/347 (24%) 89/347 (26%) 87/347 (25%) 35/347 (10%) 14/347 (4%) 38/347 (11%) 36/347 (10%) 28/347 (8%) 83/311 (27%) 96/311 (31%) 79/311 (25%) 75/311 (24%) 56/311 (18%) 32/311 (10%) 132/313 (42%) 95/313 (30%) 121/313 (39%) 92/313 (29%) 75/313 (24%) 30/313 (10%) 127/308 (41%) 81/308 (26%) 76/308 (25%) 75/308 (24%) 73/308 (24%) 30/308 (10%) 28/308 (9%) 11/308 (4%) 192/305 (63%) 55/305 (18%) 45/305 (15%) 56/305 (18%) 38/305 (12%) | 221/502 (44%) 228/502 (45%) 121/502 (24%) 78/502 (16%) 48/502 (10%) 200/499 (40%) 163/499 (33%) 89/499 (18%) 98/499 (20%) 59/499 (12%) 48/499 (10%) 172/500 (34%) 163/500 (33%) 98/500 (20%) 60/500 (12%) 28/500 (6%) 41/500 (8%) 30/500 (6%) 36/500 (7%) 165/469 (35%) 167/469 (36%) 77/469 (16%) 68/469 (14%) 66/469 (14%) 69/469 (15%) 119/253 (47%) 88/253 (35%) 77/253 (30%) 63/253 (25%) 42/253 (17%) 36/253 (14%) 104/246 (42%) 87/246 (35%) 44/246 (18%) 49/246 (20%) 37/246 (15%) 36/246 (15%) 22/246 (9%) 16/246 (7%) 159/246 (65%) 60/246 (24%) 34/246 (14%) 39/246 (16%) 17/246 (7%) | 98/154 (64%) 55/154 (36%) 34/154 (22%) 23/154 (15%) 11/154 (7%) 80/154 (52%) 30/154 (19%) 41/154 (27%) 27/154 (18%) 17/154 (11%) 10/154 (6%) 75/154 (49%) 29/154 (19%) 25/154 (16%) 10/154 (6%) 17/154 (11%) 6/154 (4%) 7/154 (5%) 8/154 (5%) 66/144 (46%) 25/144 (17%) 20/144 (14%) 26/144 (18%) 21/144 (15%) 21/144 (15%) 38/75 (51%) 16/75 (21%) 24/75 (32%) 20/75 (27%) 18/75 (24%) 8/75 (11%) 40/71 (56%) 7/71 (10%) 18/71 (25%) 15/71 (21%) 12/71 (17%) 9/71 (13%) 6/71 (8%) 5/71 (7%) 47/73 (64%) 18/73 (25%) 15/73 (21%) 9/73 (12%) 3/73 (4%) | 123/348 (35%) 173/348 (50%) 87/348 (25%) 55/348 (16%) 37/348 (11%) 120/345 (35%) 133/345 (39%) 48/345 (14%) 71/345 (21%) 42/345 (12%) 38/345 (11%) 97/346 (28%) 134/346 (39%) 73/346 (21%) 50/346 (14%) 11/346 (3%) 35/346 (10%) 23/346 (7%) 28/346 (8%) 99/325 (30%) 142/325 (44%) 57/325 (18%) 42/325 (13%) 45/325 (14%) 48/325 (15%) 81/178 (46%) 72/178 (40%) 53/178 (30%) 43/178 (24%) 24/178 (13%) 28/178 (16%) 64/175 (37%) 80/175 (46%) 26/175 (15%) 34/175 (19%) 25/175 (14%) 27/175 (15%) 16/175 (9%) 11/175 (6%) 112/173 (65%) 42/173 (24%) 19/173 (11%) 30/173 (17%) 14/173 (8%) | *1.4 (1.1, 1.8), 0.020* 1.0 (0.8, 1.3), 0.97 0.8 (0.6, 1.1), 0.15 1.1 (0.7, 1.6), 0.66 0.9 (0.6, 1.5), 0.79 *1.5 (1.1, 2.0), 0.0045* 1.3 (1.0, 1.8), 0.055 1.0 (0.7, 1.4), 0.81 *0.7 (0.5, 1.0), 0.039* 0.8 (0.5, 1.2), 0.27 1.2 (0.7, 2.0), 0.45 **1.6 (1.2, 2.2), 0.0014** *1.4 (1.0, 1.9), 0.029* 0.7 (0.5, 1.0), 0.059 1.2 (0.8, 1.9), 0.39 1.4 (0.7, 2.7), 0.31 0.7 (0.5, 1.2), 0.18 *0.6 (0.3, 0.9), 0.020* 0.9 (0.5, 1.5), 0.63 *1.5 (1.1, 2.0), 0.012* 1.2 (0.9, 1.7), 0.17 *0.6 (0.4, 0.8), 0.0023* **0.5 (0.4, 0.8), 0.00075** 0.7 (0.5, 1.1), 0.14 1.5 (1.0, 2.4), 0.072 1.2 (0.9, 1.7), 0.25 1.2 (0.9, 1.7), 0.26 *0.7 (0.5, 1.0), 0.042* 0.8 (0.5, 1.2), 0.24 *0.6 (0.4, 1.0), 0.032* 1.6 (0.9, 2.6), 0.088 1.0 (0.7, 1.5), 0.80 *1.5 (1.1, 2.2), 0.021* 0.7 (0.4, 1.0), 0.054 0.8 (0.5, 1.2), 0.22 *0.6 (0.4, 0.9), 0.011* 1.6 (0.9, 2.7), 0.078 1.0 (0.5, 1.8), 0.96 1.9 (0.9, 4.1), 0.11 1.1 (0.8, 1.5), 0.69 1.5 (1.0, 2.2), 0.069 0.9 (0.6, 1.5), 0.76 0.8 (0.5, 1.3), 0.44 *0.5 (0.3, 0.9), 0.031* | **3.1 (2.1, 4.6), 9.7e-9** *0.7 (0.4, 1.0), 0.040* 0.7 (0.5, 1.1), 0.13 1.1 (0.6, 1.8), 0.85 0.7 (0.3, 1.4), 0.31 **2.5 (1.7, 3.6), 5.8e-6** 0.7 (0.4, 1.1), 0.092 *1.6 (1.0, 2.5), 0.039* *0.6 (0.4, 1.0), 0.046* 0.7 (0.4, 1.3), 0.32 0.8 (0.4, 1.6), 0.58 **3.0 (2.0, 4.4), 8.9e-8** 0.7 (0.4, 1.1), 0.098 *0.6 (0.4, 0.9), 0.028* 0.6 (0.3, 1.3), 0.21 *2.9 (1.4, 6.1), 0.0038* *0.4 (0.1, 0.8), 0.0079* *0.4 (0.2, 0.9), 0.029* 0.7 (0.3, 1.4), 0.27 **2.3 (1.5, 3.5), 6.4e-5** *0.5 (0.3, 0.8), 0.0021* *0.5 (0.3, 0.8), 0.0049* 0.7 (0.4, 1.1), 0.15 0.8 (0.5, 1.3), 0.38 1.5 (0.8, 2.7), 0.18 1.4 (0.9, 2.3), 0.18 0.6 (0.3, 1.1), 0.12 0.8 (0.4, 1.3), 0.29 0.9 (0.5, 1.5), 0.67 1.0 (0.6, 1.8), 0.96 1.2 (0.5, 2.5), 0.70 *1.8 (1.1, 3.1), 0.021* *0.3 (0.1, 0.7), 0.0020* 1.1 (0.6, 1.9), 0.87 0.8 (0.4, 1.5), 0.60 0.7 (0.3, 1.3), 0.23 1.4 (0.6, 2.9), 0.42 1.0 (0.4, 2.3), 0.96 2.1 (0.7, 5.9), 0.18 1.1 (0.6, 1.8), 0.84 1.5 (0.8, 2.7), 0.19 1.5 (0.8, 2.8), 0.21 0.7 (0.3, 1.3), 0.24 *0.3 (0.1, 0.9), 0.035* | 1.0 (0.7, 1.3), 0.85 1.2 (0.9, 1.6), 0.27 0.8 (0.6, 1.2), 0.29 1.1 (0.7, 1.7), 0.61 1.1 (0.7, 1.7), 0.81 1.2 (0.9, 1.7), 0.24 **1.7 (1.3, 2.4), 0.00072** 0.7 (0.5, 1.1), 0.11 0.8 (0.5, 1.1), 0.11 0.8 (0.5, 1.3), 0.39 1.4 (0.8, 2.4), 0.19 1.2 (0.9, 1.7), 0.25 **1.8 (1.3, 2.5), 0.00023** 0.8 (0.6, 1.1), 0.22 1.5 (1.0, 2.4), 0.081 0.8 (0.4, 1.7), 0.55 0.9 (0.6, 1.5), 0.72 0.6 (0.4, 1.1), 0.080 1.0 (0.6, 1.7), 0.99 1.2 (0.9, 1.7), 0.29 **1.7 (1.3, 2.4), 0.00083** *0.6 (0.4, 0.9), 0.016* **0.5 (0.3, 0.7), 0.00027** 0.7 (0.5, 1.1), 0.15 1.5 (0.9, 2.4), 0.090 1.1 (0.8, 1.7), 0.47 *1.6 (1.1, 2.3), 0.024* *0.7 (0.5, 1.0), 0.048* 0.8 (0.5, 1.2), 0.22 *0.5 (0.3, 0.8), 0.0049* *1.8 (1.0, 3.0), 0.044* 0.8 (0.6, 1.2), 0.32 **2.4 (1.6, 3.5), 1.6e-5** *0.5 (0.3, 0.9), 0.010* 0.8 (0.5, 1.2), 0.22 *0.5 (0.3, 0.9), 0.013* 1.7 (1.0, 2.9), 0.064 1.0 (0.5, 1.9), 0.96 1.8 (0.8, 4.2), 0.17 1.1 (0.7, 1.6), 0.70 1.5 (0.9, 2.3), 0.10 0.7 (0.4, 1.3), 0.25 0.9 (0.6, 1.5), 0.80 0.6 (0.3, 1.2), 0.15 | **3.2 (2.2, 4.7), 4e-9** *0.6 (0.4, 0.8), 0.0036* 0.9 (0.5, 1.3), 0.49 0.9 (0.6, 1.6), 0.83 0.7 (0.3, 1.3), 0.23 **2.0 (1.4, 3.0), 0.00033** **0.4 (0.2, 0.6), 1.8e-5** **2.2 (1.4, 3.6), 0.00081** 0.8 (0.5, 1.3), 0.44 0.9 (0.5, 1.6), 0.75 0.6 (0.3, 1.1), 0.12 **2.4 (1.6, 3.6), 9e-6** **0.4 (0.2, 0.6), 7.3e-6** 0.7 (0.4, 1.2), 0.21 *0.4 (0.2, 0.8), 0.0098* **3.7 (1.7, 8.2), 0.00073** *0.4 (0.1, 0.9), 0.017* 0.7 (0.3, 1.6), 0.40 0.6 (0.3, 1.4), 0.27 **1.9 (1.3, 2.9), 0.0014** **0.3 (0.2, 0.4), 1.5e-8** 0.8 (0.4, 1.3), 0.34 1.5 (0.9, 2.5), 0.14 1.1 (0.6, 1.8), 0.80 1.0 (0.6, 1.7), 0.99 1.2 (0.7, 2.1), 0.45 *0.4 (0.2, 0.7), 0.0032* 1.1 (0.6, 2.0), 0.71 1.2 (0.6, 2.1), 0.65 *2.0 (1.0, 4.0), 0.043* 0.7 (0.3, 1.5), 0.32 *2.2 (1.3, 3.9), 0.0047* **0.1 (0.1, 0.3), 1.7e-8** 2.0 (1.0, 3.8), 0.054 1.1 (0.6, 2.2), 0.73 1.2 (0.6, 2.6), 0.57 0.8 (0.4, 1.8), 0.62 1.0 (0.3, 2.4), 0.93 1.2 (0.4, 3.3), 0.76 1.0 (0.6, 1.7), 0.94 1.0 (0.5, 1.9), 0.92 2.1 (1.0, 4.4), 0.050 0.7 (0.3, 1.5), 0.35 0.5 (0.1, 1.6), 0.30 |   Number (N) and percentage (%) of carriers of 44 HLA alleles in controls and cases, with odds ratios (ORs), 95% confidence intervals (CIs) and p-values for association with the three case definitions from univariate logistic regression models, estimated by Firth's penalised likelihood logistic regression. Associations that are significant at the 5% level are in italics, and those that are significant after correcting for multiple tests (p < 0.0015) are in bold type. The number of tests corrected for was adjusted downward from 44 to 33 to account for correlations among the tests. All cases, all classical Hodgkin lymphoma cases; EBV+ cases, EBV-positive classical Hodgkin lymphoma cases; EBV- cases, EBV-negative classical Hodgkin lymphoma cases. #A*24 includes A*24:02 and A*24:03. |
| --- | --- | --- | --- | --- | --- | --- | --- | --- | --- | --- | --- | --- | --- | --- | --- | --- | --- | --- | --- | --- | --- | --- | --- | --- | --- | --- | --- |

| **Table S5: Allele frequencies of three HLA SNPs in cases and controls** | | | | | | | | | |
| --- | --- | --- | --- | --- | --- | --- | --- | --- | --- |
|  | N minor alleles/N alleles (%) | | | |  | OR (95% CI), p-value | | | |
|  | Controls | All cases | EBV+ cases | EBV− cases |  | Cases vs controls | EBV+ cases vs controls | EBV− cases vs controls | EBV+ cases vs EBV− cases |
| rs6903608C rs2248462A rs2395185T | 206/682 (30%) 147/644 (23%) 238/678 (35%) | 350/950 (37%) 142/928 (15%) 222/946 (23%) | 68/292 (23%) 43/282 (15%) 72/294 (24%) | 282/658 (43%) 99/646 (15%) 150/652 (23%) |  | 1.3 (1.1, 1.6), p=0.0065 0.6 (0.5, 0.8), p=0.00023 0.6 (0.5, 0.7), p=5.6e-7 | 0.7 (0.5, 1.0), p=0.024 0.6 (0.4, 0.9), p=0.0095 0.6 (0.4, 0.8), p=0.0011 | 1.7 (1.4, 2.2), p=2.9e-6 0.6 (0.5, 0.8), p=0.00085 0.6 (0.4, 0.7), p=2.7e-6 | 0.4 (0.3, 0.6), p=1.3e-7 1.0 (0.7, 1.5), p=0.98 1.1 (0.8, 1.5), p=0.62 |

Number (N) and percentage (%) of minor alleles of three SNPs in controls and cases, with odds ratios (ORs), 95% confidence interval (CI) and p-values for additive association with the three case definitions from logistic regression models. All cases, all classical Hodgkin lymphoma cases; EBV+ cases, EBV-positive classical Hodgkin lymphoma cases; EBV- cases, EBV-negative classical Hodgkin lymphoma cases.

| **Table S6: Observed and expected numbers of genotypes at the seven HLA and three SNP loci, with χ^2^-tests of Hardy-Weinberg equilibrium. HLA alleles with frequency < 15% (labelled xx:xx) were pooled.** | | | | | | | | |
| --- | --- | --- | --- | --- | --- | --- | --- | --- |
| Locus | Number | Observed (expected) genotype counts | | | | | | HWE P-value |
| HLA-A | 347 | *01:01* *02:01* *03:01* *xx:xx* | *01:01* 13 (13.7) 32 (38.8) 24 (21.1) 39.5 (40.2) | *02:01*   37 (27.4) 39.5 (40.2) 66 (71.6) | *03:01*     7 (8.1) 45 (38.9) | *xx:xx*       44 (46.8) |  | 0.19 |
| HLA-C | 347 | *07:01* *07:02* *xx:xx* | *07:01* 10 (9.7) 19 (17.0) 77 (79.6) | *07:02*   10 (7.5) 63 (70.0) | *xx:xx*     168 (163.2) |  |  | 0.58 |
| HLA-B | 347 | *07:02* *08:01* *xx:xx* | *07:02* 10 (7.1) 13 (12.8) 66 (72.0) | *08:01*   6 (5.8) 65 (65.5) | *xx:xx*     187 (183.7) |  |  | 0.62 |
| HLA-DRB1 | 311 | *03:01* *15:01* *xx:xx* | *03:01* 4 (6.1) 11 (14.4) 68 (60.4) | *15:01*   7 (8.5) 78 (71.5) | *xx:xx*     143 (150.0) |  |  | 0.30 |
| HLA-DQA1 | 313 | *01:01* *01:02* *03:01* *05:01* *xx:xx* | *01:01* 12 (8.6) 10 (17.9) 20 (22.4) 30 (24.3) 25 (23.2) | *01:02*   13 (9.3) 23 (24.8) 25 (29.5) 22.5 (21.5) | *03:01*     14 (14.6) 29.5 (27.3) 25 (27.4) | *05:01*       20 (18.5) 31 (30.8) | *xx:xx*         13 (12.9) | 0.18 |
| HLA-DQB1 | 308 | *02:01*  *06:02* *xx:xx* | *02:01* 26 (19.0) 18 (23.3) 83 (91.7) | *06:02*   13 (7.2) 50 (56.3) | *xx:xx*     118 (110.5) |  |  | 0.014 |
| HLA-DPB1 | 305 | *04:01* *xx:xx* | *04:01* 68 (55.4) 124 (149.2) | *xx:xx*   113 (100.4) |  |  |  | 0.0032 |
| rs6903608 | 341 | *C* *T* | *C* 24 (31.1) 158 (143.8) | *T*   159 (166.1) |  |  |  | 0.068 |
| rs2248462 | 322 | *A* *G* | *A* 16 (16.8) 115 (113.4) | *G*   191 (191.8) |  |  |  | 0.81 |
| rs2395185 | 339 | *G* *T* | *G* 141 (142.8) 158 (154.5) | *T*   40 (41.8) |  |  |  | 0.67 |

**Table S7: Analysis of selected HLA alleles after adjustment for the effects of sex, age group, HLA-A*01:01 and A*02:01**

|  | | Cases  vs controls | | | EBV+ cases  vs controls | | | EBV- cases  vs controls | | | EBV+ cases  vs EBV- cases | | |
| --- | --- | --- | --- | --- | --- | --- | --- | --- | --- | --- | --- | --- | --- |
|  |  | Odds ratio  (95% CI) | p-value | AIC_c_ –  AIC_c carrier_ | Odds ratio  (95% CI) | p-value | AIC_c_ –  AIC_c carrier_ | Odds ratio  (95% CI) | p-value | AIC_c_ –  AIC_c carrier_ | Odds ratio  (95% CI) | p-value | AIC_c_ –  AIC_c carrier_ |
| HLA-B*08:01 | Carrier  Aadditive  Homozygote | 1.50 (1.03, 2.19) 1.71 (1.24, 2.36) 4.46 (1.93, 11.88) | 0.037  0.00093  0.00024 | -  -6.6  -9.1 | 1.70 (1.06, 2.74) 1.92 (1.30, 2.85) 4.65 (1.86, 13.09) | 0.029  0.0010  0.00078 | -  -6.0  -6.6 | -  -  - | -  -  - | -  -  - | 1.01 (0.6, 1.7)  -  - | 0.97  -  - | -  -  - |
| HLA-B*35:01 | Carrier | 0.99 (0.59, 1.68) | 0.97 | - | 0.74 (0.30, 1.64) | 0.47 | - | - | - | - | 0.90 (0.37, 1.99) | 0.80 | - |
| HLA-DRB1*15:01 | Carrier  Additive  Homozygote | 1.41 (0.97, 2.06)  -  - | 0.27  -  - | -  -  - | 0.48 (0.28, 0.79) 0.57 (0.35, 0.89) 1.40 (0.33, 4.94) | 0.0036  0.013  0.62 | -  2.3  8.3 | 1.65 (1.18, 2.33) 1.70 (1.28, 2.27) 4.14 (1.85, 10.42) | 0.0038  0.00020  0.00035 | -  -5.4  -4.4 | 0.34 (0.20, 0.56) 0.41 (0.26, 0.62) 0.36 (0.09, 1.04) | 1.2e-5  1.5e-5  0.060 | -  0.4  15.6 |
| HLA-DQB1*06:02 | Carrier  Additive  Homozygote | 1.41 (0.97, 2.06)  -  - | 0.071  -  - | -  -  - | 0.36 (0.15, 0.77) 0.38 (0.16, 0.76) 0.22 (0.00, 1.70) | 0.0073  0.0045  0.18 | -  -0.8  5.5 | 2.08 (1.38, 3.12) 2.00 (1.49, 2.71) 4.97 (2.51, 10.34) | 0.00042  3.5e-6  2.6e-6 | -  -9.1  -9.7 | 0.19 (0.08, 0.43) 0.24 (0.10, 0.47) 0.04 (0.00, 0.28) | 2.1e-5  2.5e-6  0.00013 | -  -4.1  3.5 |
| HLA-DPB1*02:01 | Carrier  Homozygote | 0.84 (0.51, 1.37)  - | 0.49  - | -  - | 1.31 (0.64, 2.56)  - | 0.44  - | -  - | 0.62 (0.33, 1.10)  - | 0.10  - | -  - | 2.47 (1.04, 5.84) 2.58 (1.24, 5.63) | 0.040  0.011 | -  -2.4 |
| HLA-DPB1*03:01 | Carrier | 1.44 (0.94, 2.19)) | 0.090 | - | 1.61 (0.84, 2.99) | 0.15 | - | 1.43 (0.89, 2.28) | 0.14 | - | 1.33 (0.66, 2.62) | 0.42 | - |

Odds ratios (95% confidence intervals [CI]) and p-values for association between selected HLA alleles and the four case-control outcomes were estimated using Firth's penalised likelihood logistic regression. HLA-B*08:01 and B*35:01 were selected because they elicit immunodominant EBV-specific responses and therefore were included only in analyses of EBV-positive cases. DRB1*15:01, DQB1*06:02, DPB1*02:01 and DPB1*03:01 were selected because they have previously been associated with disease risk. To avoid multiple testing across inheritance models, a carrier model was tested initially, and only if this proved significant (p < 0.05) were alternative (additive and homozygote) models considered. A reduction of at least two AICc units relative to the carrier model indicated evidence in favour of alternative model(s). All analyses were adjusted for the effects of sex, age group, and the additive effects of HLA-A*01:01 and A*02:01 alleles. All cases, all classical Hodgkin lymphoma cases; EBV+ cases, EBV-positive classical Hodgkin lymphoma cases; EBV- cases, EBV-negative classical Hodgkin lymphoma cases; corrected Akaike information criterion, AIC; - , not tested.

**Table S8: Allele frequencies within cases and controls and univariate odds ratios for associations between HLA alleles and classical Hodgkin lymphoma, with alleles grouped according to Figure S**1 of Huang et al (2012)

|  |  | All cases  vs Controls | | |  | EBV+ cases  vs Controls | | |  | EBV- cases  vs Controls | | |
| --- | --- | --- | --- | --- | --- | --- | --- | --- | --- | --- | --- | --- |
|  |  | N | Allele freq. (%) | OR (99% CI)  p value |  | N | Allele freq. (%) | OR (99% CI)  p value |  | N | Allele freq. (%) | OR (99% CI)  p value |
| A1 | Control Case | 347 502 | 19.9 26.4 | 1.41 (1.05, 1.92) p=0.0025 |  | 347 154 | 19.9 40.6 | **2.71 (1.82, 4.09) p = 4.1 x 10^-11^** |  | 347 348 | 19.9 20.1 | 1.01 (0.72, 1.43) p=0.92 |
| A2 | Control Case | 347 502 | 29.1 27.8 | 0.94 (0.71, 1.24) p=0.56 |  | 347 154 | 29.1 20.5 | **0.64 (0.42, 0.97) p=0.0050** |  | 347 348 | 29.1 31.0 | 1.09 (0.81, 1.46) p=0.45 |
| B5 | Control Case | 347 500 | 3.9 4.0 | **1.02 (0.55, 1.96) p=0.93** |  | 347 154 | 3.9 3.6 | 0.64 (0.42, 0.97) p=0.87 |  | 347 346 | 3.9 4.2 | 1.08 (0.54, 2.16) p=0.78 |
| B37 | Control Case | 347 500 | 2.0 2.9 | **1.41 (0.64, 3.41) p=0.27** |  | 347 154 | 2.0 5.8 | **2.91 (1.17, 7.62) p=0.0026** |  | 347 346 | 2.0 1.6 | 0.79 (0.27, 2.23) p=0.55 |
| DR2 | Control Case | 311 469 | 17.2 22.1 | **1.33 (0.96, 1.86) p=0.023** |  | 311 144 | 17.2 11.5 | 0.63 (0.36, 1.07) p=0.026 |  | 311 325 | 17.2 26.8 | **1.71 (1.21, 2.46) p = 5.8 x 10^-5^** |
| DR5 | Control Case | 311 469 | 9.0 8.2 | **0.90 (0.57, 1.45) p=0.58** |  | 311 144 | 9.0 4.5 | 0.48 (0.20, 1.04) p=0.015 |  | 311 325 | 9.0 9.8 | **1.10 (0.67, 1.82) p=0.61** |
| DR7 | Control Case | 311 469 | 13.0 7.6 | **0.55 (0.35, 0.86) p=0.00050** |  | 311 144 | 13.0 10.1 | 0.77 (0.42, 1.33) p=0.22 |  | 311 325 | 13.0 6.5 | **0.46 (0.27, 0.76) p = 7 x 10^-5^** |
| DR10 | Control Case | 311 469 | 1.0 1.2 | 1.15 (0.36, 4.39) p=0.76 |  | 311 144 | 1.0 2.4 | **2.23 (0.59, 9.43) p=0.11** |  | 311 325 | 1.0 0.6 | 0.66 (0.11, 3.25) p=0.49 |

Findings reported by Huang et al (2012)^2^ were tested following grouping of alleles into broad HLA serotypes: B5 = B*51, B*52, B*51:02 plus B*51:03; B37 = B*37; DR2 = DRB1*15 plus DRB1*16; DR5 = DRB1*11 plus DRB1*12; DR7 = DRB1*07 and DR10 = DRB1*10. For consistency 99% confidence intervals (CIs) are reported. OR, odds ratio; all cases, all classical Hodgkin lymphoma cases; EBV+ cases, EBV-positive cHL cases; EBV- cases, EBV-negative cHL cases. ORs, CIs and p values shown in bold indicate associations that were significant in Huang et al (2012).

**Table S9: Allele selection model for EBV-positive classical Hodgkin lymphoma without inclusion of SNPs, adjusted for sex and age group**

| Variable |  | PPA |  | Odds ratio  (95% CI) |  | p-value |
| --- | --- | --- | --- | --- | --- | --- |
| HLA-A*01:01 additive# |  | 97% |  | 2.05 (1.41, 3.01) |  | 0.00018 |
| HLA-A*02:01 additive# |  | 8% |  | 0.99 (0.67, 1.44) |  | 0.94 |
| HLA-B*37:01 carrier |  | 62% |  | 3.01 (1.32, 7.06) |  | 0.0086 |
| HLA-DRB1*03:01 homozygote |  | 58% |  | 5.78 (2.04, 19.86) |  | 0.00068 |
| HLA-DRB1*15:01 carrier |  | 69% |  | 0.50 (0.29, 0.84) |  | 0.0076 |
| HLA-DPB1*01:01 additive◊ |  | ▲ |  | 0.21 (0.05, 0.54) |  | 0.00033 |

Bayesian variable selection modelling was performed on EBV-positive Hodgkin lymphoma cases versus controls with inclusion of forty-four HLA alleles; alleles with a posterior probability of association (PPA) ≥ 50% were selected and refitted in a Firth logistic regression model to generate odds ratios and confidence intervals (CIs). ▲Both carrier (PPA 61%) and homozygote (PPA 58%) effects of DPB*01:01 were selected, but the model fit was improved by replacing both these effects with a single additive effect, shown here. #, adjustment variable, not subject to variable selection; ◊data derived from analysis of smaller data set.

**Table S10: Addition of the A*01:01-C*06:02-B*37:01 haplotype to the final model for EBV-positive classical Hodgkin lymphoma, adjusted for sex and age group**

| Variable |  | Odds ratio (95% CI) |  | p-value |
| --- | --- | --- | --- | --- |
| HLA-A*01:01 additive |  | 2.60 (1.82, 3.77) |  | 1 x 10^-7^ |
| HLA-A*02:01 additive |  | 0.99 (0.67, 1.44) |  | 0.94 |
| HLA-B*37:01 carrier |  | 9.48 (1.44, 108.51) |  | 0.019 |
| HLA-DRB1*15:01 carrier |  | 0.46 (0.27, 0.77) |  | 0.0026 |
| Hap A*01:01-C*06:02-B*37:01 carrier |  | 0.18 (0.01, 1.69) |  | 0.13 |

Odds ratios, 95% confidence intervals (CIs) and p-values for alleles selected from HLA A, C, B and DRB1 (Table 3), and for the A*01:01-C*06:02-B*37:01 haplotype, estimated from a Firth logistic regression model where the outcome was EBV-positive classical Hodgkin lymphoma versus controls, fitted on the full data set. Estimates for adjustment variables (additive effects of A*01:01 and A*02:01) are also presented. The A*01:01-C*06:02-B*37:01 haplotype was included only as a carrier effect as no subjects were homozygous for this haplotype. Hap, haplotype.

**Table S11: Addition of infectious mononucleosis to the final model for EBV-positive classical Hodgkin lymphoma, adjusted for sex and age group**

| Variable |  | Odds ratio  (95% CI) |  | p-value |
| --- | --- | --- | --- | --- |
| Self-reported IM |  | 4.51(1.44, 14.04) |  | 0.011 |
| HLA-A*01:01 additive |  | 2.48 (1.53, 4.07) |  | 0.00022 |
| HLA-A*02:01 additive |  | 1.14 (0.66, 1.90) |  | 0.63 |
| HLA-A*02:01 additive x IM |  | 0.19 (0.00, 2.61) |  | 0.24 |
| HLA-B*37:01 carrier |  | 3.33 (1.16, 9.38) |  | 0.026 |
| HLA-DRB1*15:01 carrier |  | 0.26 (0.11, 0.55) |  | 0.00025 |
| HLA-DPB1*01:01 carrier |  | 0.19 (0.04, 0.56) |  | 0.0017 |

Odds ratios, 95% confidence intervals (CIs) and p-values for alleles selected in the final analysis of EBV-positive classical Hodgkin lymphoma (Table 4) plus self-reported history of infectious mononucleosis (IM) were estimated from a Firth logistic regression model of EBV-positive classical Hodgkin lymphoma fitted on the smaller dataset. Estimates are also presented for the adjustment variables A*01:01 and A*02:01 and the previously reported interaction between A*02:01 and IM; analysis was also adjusted for sex and age group. In a similar analysis performed on the larger dataset (HLA A, C, B, and DRB1 alleles) self-reported IM had an odds ratio of 3.19; 95% CI 1.15-8.71; p=0.026.

**Table S12: Association between A*02:01 and EBV-positive Hodgkin lymphoma in case: control and case series analysis with adjustment for the effects of sex, age group and A*01:01**

|  |  | Cases versus controls | | EBV+ cases versus EBV- cases | |
| --- | --- | --- | --- | --- | --- |
| Variable | Level | Odds ratio (95% CI) | P-value | Odds ratio (95% CI) | P-value |
| Sex | Female Male | 1.00 (reference) 1.50 (0.99, 2.29) | 0.054 | 1.00 (reference) 2.10 (1.38, 3.23) | 0.00047 |
| Age group (years) | 15-34 35-49 50+ | 1.00 (reference) 0.78 (0.45, 1.32) 1.00 (0.64, 1.57) | 0.59 | 1.00 (reference) 1.36 (0.79, 2.31) 2.78 (1.74, 4.46) | 9.7×10^-5^ |
| HLA-A*01:01 additive |  | 2.58 (1.86, 3.62) | 7.4×10^-9^ | 2.32 (1.67, 3.26) | 4.1×10^-7^ |
| HLA-A*02:01 additive |  | 0.90 (0.63, 1.28) | 0.57 | 0.83 (0.57, 1.22) | 0.35 |

EBV+ cases, EBV-positive cases; EBV- cases, EBV-negative cases; CI, confidence interval.

The above cases constituted the UK series included in the case series analysis reported by Hjalgrim et al (2010) ^10^. In this analysis of EBV-positive cases versus EBV-negative cases, which also included cases from Denmark and Sweden, the effect of HLA-A*02:01 was significant and there was no evidence of heterogeneity by country.

**Table S13: Allele selection model for EBV-negative Hodgkin lymphoma cases without inclusion of SNPs, adjusted for sex and age group**

| Variable |  | PPA |  | Odds ratio  (95% CI) |  | p-value |
| --- | --- | --- | --- | --- | --- | --- |
| HLA-DRB1*07:01 homozygote |  | 64% |  | 0.06 (0.00, 0.58) |  | 0.010 |
| HLA-DQB1*06:02 homozygote◊ |  | 67% |  | 5.68 (2.80, 12.17) |  | 7.4 x 10^-7^ |

Bayesian variable selection modelling was performed on EBV-negative Hodgkin lymphoma cases versus controls with inclusion of forty-four HLA alleles; alleles with a posterior probability of association (PPA) ≥ 50% were selected and refitted in a Firth logistic regression model to generate odds ratios and confidence intervals (CIs). #, adjustment variable, not subject to variable selection; ◊data derived from analysis of smaller data set.

**Table S14: Allele selection model for EBV-negative classical Hodgkin lymphoma cases with addition of the rs6903608C-DRB1*15:01-DQB1*06:02 haplotype, adjusted for sex and age group**

| Variable |  | Odds ratio (95% CI) |  | p-value |
| --- | --- | --- | --- | --- |
| HLA-B*15:01 carrier |  | 2.33 (1.27, 4.31) |  | 0.0068 |
| HLA-DRB1*03:01 homozygote |  | 7.42 (2.25, 27.73) |  | 0.0010 |
| HLA-DQB1*03:03 carrier |  | 3.53 (1.37, 9.17) |  | 0.0092 |
| rs6903608C homozygote |  | 3.20 (1.48, 7.08) |  | 0.0030 |
| Hap rs6903608C-DRB1*15:01-DQB1*06:02 homozygote |  | 2.67 (0.83, 9.24) |  | 0.099 |
| Hap rs6903608C-DRB1*15:01-DQB1*06:02 carrier |  | 1.53 (0.92, 2.50) |  | 0.098 |

Odds ratios, 95% confidence intervals (CIs) and p-values for alleles selected from HLA A, C, B and DRB1 (Table 5), and for the rs6903608C-DRB1*15:01-DQB1*06:02 haplotype, estimated from a Firth logistic regression model where the outcome was EBV-negative classical Hodgkin lymphoma versus controls, fitted on the full data set. Estimates for adjustment variables (additive effects of A*01:01 and A*02:01) are also presented. Hap, haplotype.

**Table S15: Allele selection model for all classical Hodgkin lymphoma cases, adjusted for sex and age group**

|  |  | PPA |  | Odds ratio  (95% CI) |  | p-value |
| --- | --- | --- | --- | --- | --- | --- |
| HLA-A*01:01 additive# |  | 30% |  | 1.42 (1.07, 1.88) |  | 0.014 |
| HLA-A*02:01 additive# |  | 9% |  | 0.98 (0.76, 1.26) |  | 0.90 |
| rs6903608 CC |  | 74% |  | 2.93 (1.79, 4.97) |  | 1.2 x 10^-5^ |
| HLA-DRB1*03:01 homozygote |  | 70% |  | 5.55 (2.15, 17.94) |  | 0.00017 |
| HLA-DQB1*03:03 carrier◊ |  | 78% |  | 2.94 (1.30, 6.93) |  | 0.0094 |
| HLA-DPB1*01:01 carrier◊ |  | 64% |  | 0.43 (0.21, 0.83) |  | 0.012 |

Bayesian variable selection modelling was performed on all cases versus controls, with inclusion of forty-four HLA alleles and three selected HLA SNPs; alleles with a posterior probability of association (PPA) ≥ 50% were selected and refitted in a Firth logistic regression model to generate odds ratios and confidence intervals (CIs). #, adjustment variable, not subject to variable selection; ◊data derived from analysis of smaller dataset.

**Table S16: Allele selection model for all classical Hodgkin lymphoma cases without inclusion of SNPs, adjusted for sex and age group**

|  |  | PPA |  | Odds ratio  (95% CI) |  | p-value |
| --- | --- | --- | --- | --- | --- | --- |
| HLA-A*01:01 additive# |  | 37% |  | 1.39 (1.05, 1.85) |  | 0.020 |
| HLA-A*02:01 additive# |  | 8% |  | 1.04 (0.81, 1.34) |  | 0.75 |
| HLA-DRB1*03:01 homozygote |  | 70% |  | 4.21 (1.62, 13.73) |  | 0.0022 |
| HLA-DRB1*04:01 carrier |  | 55% |  | 0.61 (0.41, 0.88) |  | 0.0094 |
| HLA-DRB1*07:01 carrier |  | 57% |  | 0.59 (0.40, 0.87) |  | 0.0027 |
| HLA-DRB1*15:01 homozygote |  | 57% |  | 2.59 (1.18, 6.45) |  | 0.017 |
| HLA-DPB1*01:01 carrier◊ |  | 51% |  | 0.43 (0.21, 0.82) |  | 0.010 |

Bayesian variable selection modelling was performed on all cases versus controls, with inclusion of forty-four HLA alleles; alleles with a posterior probability of association (PPA) ≥ 50% were selected and refitted in a Firth logistic regression model to generate odds ratios and confidence intervals (CIs). #, adjustment variable, not subject to variable selection; ◊data derived from analysis of smaller dataset.

**Table S17: Allele selection model for EBV-positive versus EBV-negative Hodgkin lymphoma cases**

|  |  | Level |  | PPA |  | Odds ratio  (95% CI) |  | Df |  | p-value |
| --- | --- | --- | --- | --- | --- | --- | --- | --- | --- | --- |
| Sex# |  | Male |  | 99% |  | 2.16 (1.38, 3.41) |  | 1 |  | 0.00067 |
| Age group (years)# |  | 35-49  50+ |  | 15%  71% |  | 1.54 (0.87, 2.72)  2.55 (1.55, 4.23) |  | 2 |  | 0.0011 |
| HLA-A*01:01 additive# |  |  |  | 77% |  | 1.81 (1.26, 2.62) |  | 1 |  | 0.0013 |
| HLA-A*02:01 additive# |  |  |  | 16% |  | 0.85 (0.57, 1.27) |  | 1 |  | 0.43 |
| HLA-B*27:05 carrier |  |  |  | 50% |  | 0.47 (0.17, 1.12) |  | 1 |  | 0.092 |
| HLA-B*37:01 carrier |  |  |  | 68% |  | 3.66 (1.53, 9.14) |  | 1 |  | 0.023 |
| HLA-DRB1*15:01 carrier |  |  |  | 95% |  | 0.31 (0.18, 0.51) |  | 1 |  | 2.6 x 10^-6^ |
| HLA-DQA1*01:02 carrier◊ |  |  |  | 59% |  | 7.38 (1.40, 77.83) |  | 1 |  | 0.016 |

Bayesian variable selection modelling was performed on EBV-positive cases versus EBV-negative cases, with inclusion of forty-four HLA alleles and three selected HLA SNPs; alleles with a posterior probability of association (PPA) ≥ 50% were selected and refitted in a Firth logistic regression model to generate odds ratios and confidence intervals (CIs). Df, degrees of freedom; #, adjustment variable, not subject to variable selection; ◊data derived from analysis of smaller dataset.

**Table S18: Allele selection model for EBV-positive versus EBV-negative Hodgkin lymphoma cases without inclusion of SNPs**

| Variable |  | Level |  | PPA |  | Odds ratio  (95% CI) |  | Df |  | p-value |
| --- | --- | --- | --- | --- | --- | --- | --- | --- | --- | --- |
| Sex# |  | Male |  | 97% |  | 2.18 (1.41,3.43) |  | 1 |  | 0.00046 |
| Age group (years)# |  | 35-49  50+ |  | 15%  94% |  | 1.47 (0.83, 2.58)  2.47 (1.51, 4.06) |  | 2 |  | 0.0015 |
| HLA-A*01:01 additive# |  |  |  | 98% |  | 2.09 (1.47, 2.99) |  | 1 |  | 3 x 10^-5^ |
| HLA-A*02:01 additive# |  |  |  | 10% |  | 0.85 (0.57, 1.26) |  | 1 |  | 0.42 |
| HLA-DRB1*15:01 carrier |  |  |  | 100% |  | 0.34 (0.20, 0.56) |  | 1 |  | 1.2 x 10^-5^ |
| HLA-DQA1*01:02 carrier◊ |  |  |  | 62% |  | 9.89 (1.93, 101.98) |  | 1 |  | 0.0042 |

Bayesian variable selection modelling was performed on EBV-positive cases versus EBV-negative cases, with inclusion of forty-four HLA alleles; alleles with a posterior probability of association (PPA) ≥ 50% were selected and refitted in a Firth logistic regression model to generate odds ratios and confidence intervals (CIs). Df, degrees of freedom; #, adjustment variable, not subject to variable selection; ◊data derived from analysis of smaller dataset.


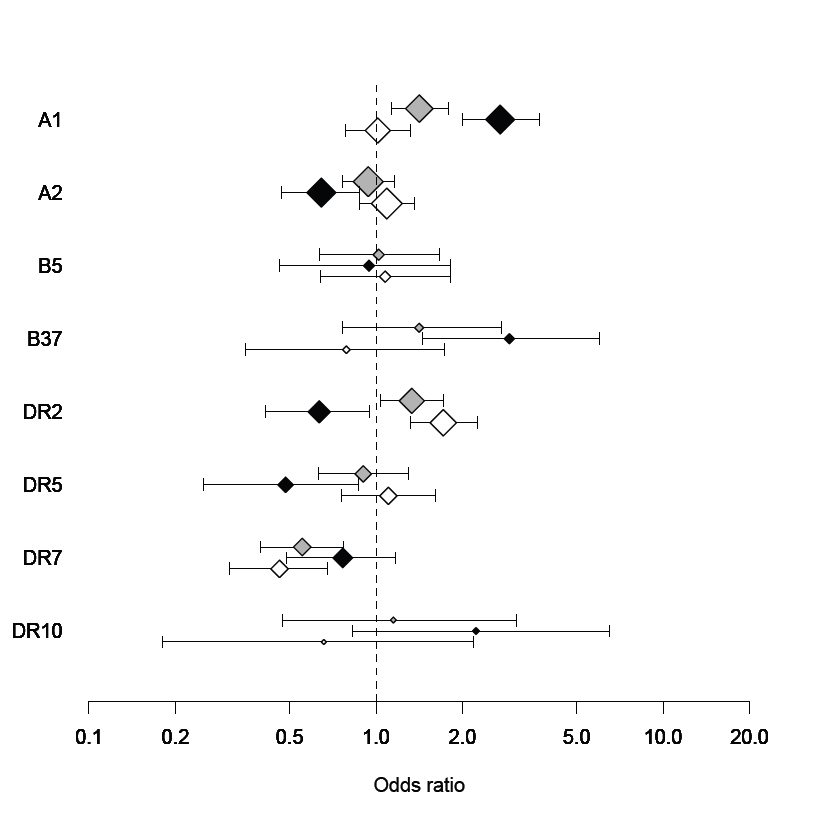


**Figure S1: Unadjusted analyses confirm main findings reported by Huang et al (2012)^2^**

Forest plot showing odds ratios and 95% confidence intervals of unadjusted analyses of alleles showing significant or ‘nearly significant’ differences in the study reported by Huang et al (PLoS.ONE. 2012;7:e39986). Controls versus ‘all cases’, grey diamonds; controls versus EBV-positive cases, black diamonds; and controls versus EBV-negative cases, white diamonds. The size of the diamonds reflects the allele frequency.

These data corroborate: an increased frequency of A1 alleles in EBV-positive cases; a decreased frequency of A2 alleles in EBV-positive cases; an increased frequency of B37 alleles in EBV-positive cases; an increased frequency of DR2 alleles in ‘all cases’ and EBV-negative cases; and a decreased frequency of DR7 alleles in ‘all cases’. We did not detect a significantly increased frequency of B5 alleles in ‘all cases’; an increased frequency of DR5 in ‘all cases’ or EBV-negative cases; or an increased frequency of DR10 alleles in EBV-positive cases, although the direction of risk was consistent with Huang et al (2012)
^2^. We did detect a significantly decreased frequency of DR2 alleles in EBV-positive cases; a decreased frequency of DR5 alleles in EBV-positive cases; and a decreased frequency of DR7 alleles in EBV-negative cases.
